# Hepatic GPR75 exacerbates MASH through GNAI2-dependent signaling

**DOI:** 10.1097/HEP.0000000000001706

**Published:** 2026-02-03

**Authors:** Xule Yang, Na Yang, Zhihao Cheng, Rui Zhang, Hui Fan, Dongsheng Liu, Jiye Aa, Guangji Wang, Yuan Xie

**Affiliations:** 1 Key Laboratory of Drug Metabolism and Pharmacokinetics, State Key Laboratory of Natural Medicines, China Pharmaceutical University, Nanjing, China; 2 China Pharmaceutical University Nanjing Drum Tower Hospital, Nanjing, China; 3 Research Unit of PK-PD Based Bioactive Components and Pharmacodynamic Target Discovery of Natural Medicine of Chinese Academy of Medical Sciences, China Pharmaceutical University, Nanjing, China

**Keywords:** de novo lipogenesis, GNAI2, GPR75, MASH, VPS35

## Abstract

**Background and Aims:**

Metabolic dysfunction–associated steatotic liver disease (MASLD), including its more severe form, metabolic dysfunction–associated steatohepatitis (MASH), is increasingly recognized as a critical global health challenge. This study investigates the role of hepatic GPR75 in MASH progression.

**Approach and Results:**

Although GPR75 is not abundantly expressed in the liver in healthy individuals, its protein levels significantly increase during MASH. Depletion of *Gpr75* in either the whole liver or specifically in hepatocytes protected mice from diet-induced hepatic steatosis, while hepatocyte-specific overexpression of *Gpr75* exacerbated diet-induced MASH and liver fibrosis. The deficiency of hepatic *Gpr75* activated the GNAI2-cAMP-PKA signaling pathway in the livers of MASLD mice, reducing SREBP-1c maturation and de novo lipogenesis. Mechanistically, VPS35 stabilized GPR75 by recycling it to the hepatocyte membrane, thereby decreasing its degradation during MASH progression.

**Conclusions:**

This study demonstrates that GPR75 serves as a novel regulator of MASLD/MASH by modulating hepatic fatty acid metabolism. These findings suggest that GPR75 suppression may represent a potential therapeutic strategy for MASLD/MASH treatment.

## Graphical abstract

**Figure:**
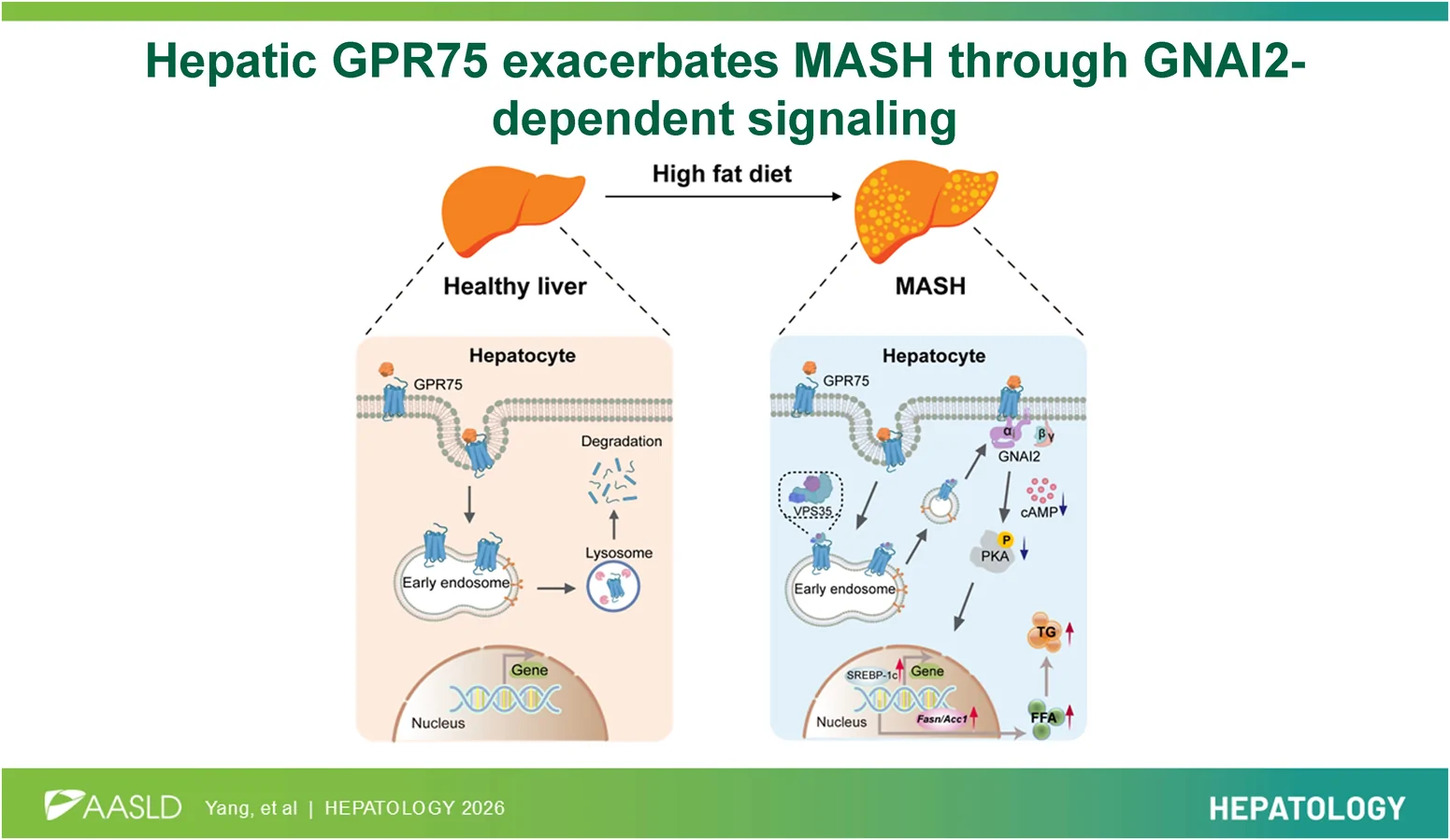

## INTRODUCTION

Metabolic dysfunction–associated steatohepatitis (MASH; previously termed nonalcoholic steatohepatitis or NASH), the hepatic manifestation of the metabolic syndrome, is now the leading etiology of chronic liver disease. A key pathogenic feature is dysregulated hepatic lipid metabolism, involving synthesis, storage, mobilization, and catabolism.^1–4^ Hepatic steatosis, an early hallmark of MASLD development, results from compensatory partitioning of excess free fatty acids—derived from elevated circulatory influx or de novo lipogenesis—into triacylglycerol within cytosolic lipid droplets.^4–6^ However, prolonged intrahepatic triacylglycerol storage can promote hepatic insulin resistance and injury via lipid peroxidation, driving the inflammation and fibrosis associated with MASH.^7^ Over the past decade, the global prevalence of MASLD has markedly increased; however, pharmacological treatments for this disease remain limited.

G-protein–coupled receptor 75 (GPR75), an orphan receptor of the GPCR family, is widely expressed in human tissues, including the brain, retina, kidney, heart, and prostate.^8–10^ Human genetic studies identified variants in the *GPR75* gene that are associated with reduced body fat and lower BMI.^11^ Consistent with human data, deletion of *Gpr75* in mice confers protection against high-fat diet (HFD)-induced obesity, improves insulin sensitivity, and enhances glycemic control.^9,11^ Furthermore, analysis of whole-exome sequencing data from 428,719 UK Biobank participants linked GPR75 variants to a lower risk of hepatic steatosis, while genetic inactivation of *Gpr75* in mice protected against NAFLD and reduced fat accumulation.^9^ These findings collectively suggest that GPR75, which is predominantly expressed mainly in the brain, influences obesity and hepatic steatosis by regulating food intake. However, the mechanism by which GPR75 regulates MASH remains poorly understood, and it is unclear whether GPR75 directly regulates lipid accumulation in the liver and adipose tissue.

Recently, RANTES (CCL5) and 20-hydroxyeicosatetraenoic acid (20-HETE) have been identified as endogenous ligands for GPR75.^12^ CCL5 plays an important role in the progression of hepatic inflammation and fibrosis.^13–16^ Arachidonic acid is metabolized by the cytochrome P450 enzymes of the 4A and 4F families to form (20-HETE, which is essential for activating the GPR75 receptor, leading to vasoconstriction and cell proliferation.^17^ 20-HETE has been shown to promote insulin resistance in mice fed an HFD, which can be prevented by a 20-HETE receptor blocker.^18^ The chemokine CCL5 activates GPR75 via a Gqα protein-mediated signal transduction pathway in cells overexpressing GPR75, pancreatic islets, and neuroblastoma cell lines.^19–21^ Researchers also observe constitutive GPR75 Gi-arrestin and β-arrestin coupling.^9,22^

While genetic disruptions that promote leanness can paradoxically increase fatty liver risk, the hepatic role of GPR75 remains unexplored. To address this gap, we investigated the function of GPR75 in MASLD. In this study, we reported that hepatocyte-specific knockout of *Gpr75* (*Gpr75* ^*fl/fl*^ *TBG* ^Cre^) attenuated hepatic lipid accumulation, whereas its overexpressed (OE) exacerbated MASH in HFD-induced mice. Then, we identified GNAI2 as GPR75-coupled G-protein and revealed that GPR75 suppressed the expression of SREBPs (SREBP1c/2) via GNAI2-cAMP-PKA pathway, leading to the inhibition of DNL and inflammation. Ultimately, we demonstrated that the protein level of GPR75 was affected by VPS35, an important component of the retromer chaperone to escort GPR75 back to the cell membrane and maintains its protein level. Our study also presents the GPR75-GNAI2 pathway as a potential therapeutic target in MASH.

## METHODS

### Animal studies

All animal protocols were approved by the Animal Ethics Committee of China Pharmaceutical University (License No. 2024-09-046) and conducted in accordance with the ARRIVE guidelines (Animals in Research: Reporting In Vivo Experiments). C57BL/6J mice were housed 3–5 per cage under a 12-hour light/12-hour dark cycle at a controlled temperature (20–22 °C) with free access to food and water. For experiments, 6- to 8-week-old male mice were used, and littermate mice served as controls across all genotypes. *Gpr75* flox/flox mice (C57BL/6Smoc-*Gpr75* ^*em1(flox)Smoc*^, Cat#: NM-CKO-2112218) with comparable body weights were randomly allocated into 2 groups. Each group received a tail vein injection of either pAAV8-TBG-NLS (control) or pAAV8-TBG-NLS-Cre-3×Flag-WPRE (OBio Technology) at a dose of 2×10¹¹ GC per mouse to generate hepatocyte-specific *Gpr75* knockout mice. Detailed methods are provided in Supplemental Materials and Methods, http://links.lww.com/HEP/K371. The primer or oligonucleotide sequences are shown in Supplemental Table S1, http://links.lww.com/HEP/K371.

### Human samples

Human liver sections were obtained from BioAiTech. These samples included normal liver tissue and liver tissue exhibiting MASH or fibrosis adjacent to HCC. The vendor supplied clinical information for all samples, including sex, age, and pathological diagnosis (Supplemental Table S2, http://links.lww.com/HEP/K371). The collection and use of these human liver samples were approved by the Ethics Committee of Changsha Yaxiang Biotechnology Co., Ltd. (Approval No. Csya2024007) and were conducted in strict accordance with the principles of the Declaration of Helsinki. Written informed consent was acquired from all participants or their legal guardians.

### Statistical analyzes

Data are presented as mean ± SEM. Statistical analyses were performed using GraphPad Prism version 9 (GraphPad Software). For comparisons between 2 groups, an unpaired 2-tailed Student *t* test was used. For comparisons across more than 2 groups, 1-way ANOVA was employed. Significant differences among groups are indicated as *p<0.05, **p<0.01, ***p<0.001, ****p<0.0001, ns, no statistical significance.

Additional details are described in the Supplemental information, http://links.lww.com/HEP/K371.

## RESULTS

### Hepatic GPR75 protein expression is upregulated in patients with MASH and mice livers

A large-scale exome sequencing of 640,000 subjects identified an association between loss-of-function GPR75 variants and protection against obesity.^11^ It has been demonstrated that GPR75 is highly expressed in the mouse brain but shows low mRNA expression in the liver. Furthermore, based on Gene Expression Omnibus (GEO) datasets, no significant differences in GPR75 mRNA levels were observed in either patients with MASH or mouse livers compared to normal controls (Figure 1A). However, immunohistochemistry staining analysis suggested that the protein levels of GPR75 gradually increase from MASH to liver fibrosis in human hepatic samples (Figure 1B). Similarly, no significant change in mRNA expression of hepatic *Gpr75* was observed in mice fed a HFD or a choline-deficient, L-amino acid-defined high-fat diet (CDAHFD) using multiple qRT-PCR assays (Figure 1C, D, Supplemental Figure S1A, http://links.lww.com/HEP/K371). To further evaluate GPR75 protein expression in the liver by western blotting, we used mouse brain samples as a positive control and validated the specificity of the antibody using a GPR75 antibody blocking peptide (Supplemental Figure S1B, http://links.lww.com/HEP/K371). The upregulation of GPR75 protein level was observed in the livers of mice fed with HFD and CDAHFD diets, indicating that a post-transcriptional modification of GPR75 in MASH (Figures 1C, D). The immunofluorescence staining confirmed this result, further validating an increase in liver GPR75 protein during MASH progression (Figure 1E, F).

**FIGURE 1:**
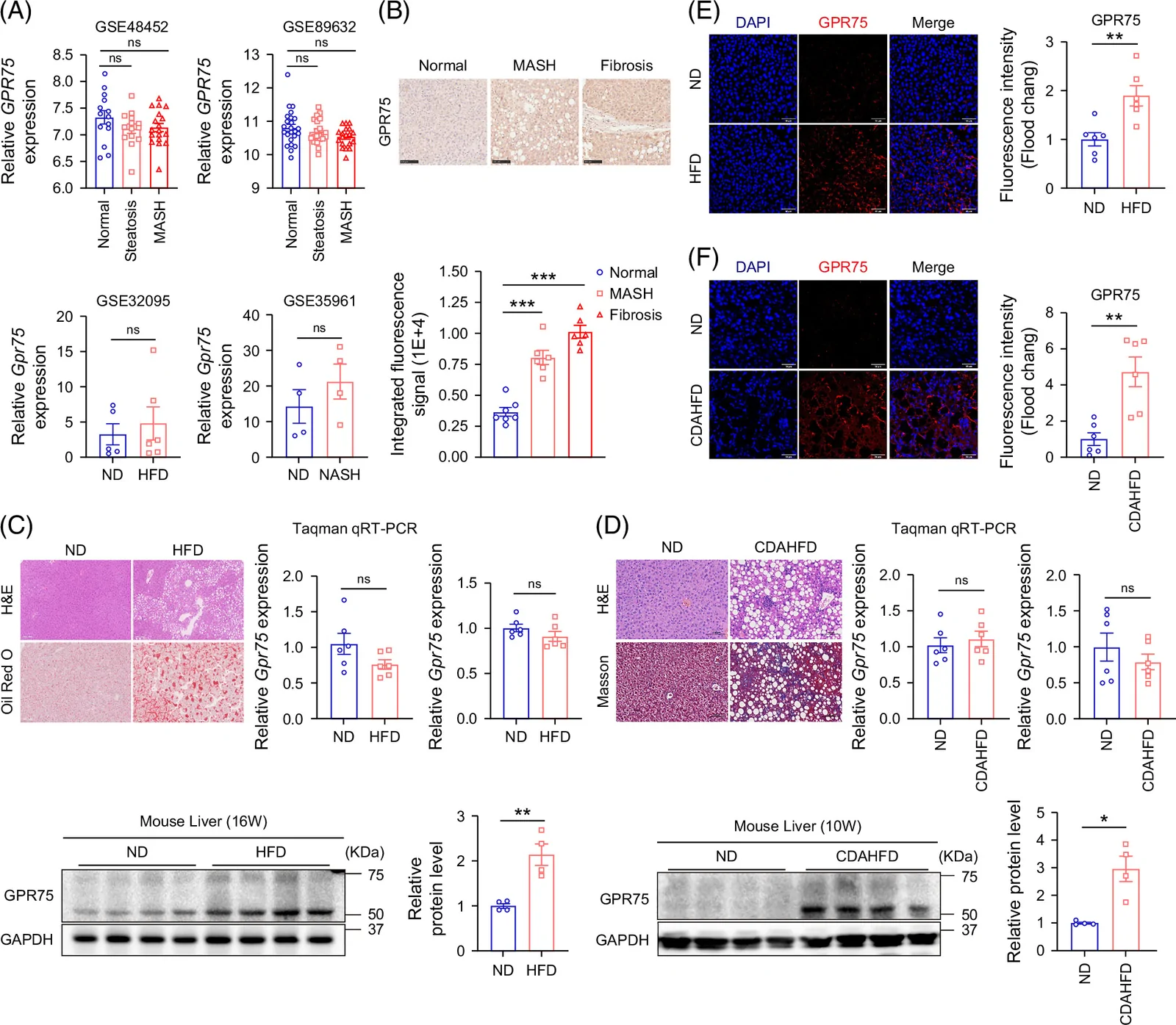
Hepatic GPR75 protein expression is upregulated in patient with MASH and mice livers. (A) Gene expression analysis of *GPR75* mRNA in publicly available datasets from the Gene Expression Omnibus (GEO) database (GSE48452, GSE89632, GSE32095, GSE35961). (B) Immunohistochemical staining of GPR75 in liver sections from human controls (n=7) and patients with MASH and fibrosis (n=6). Positive staining areas were quantified using ImageJ software. Scale bar: 100 μm. (C) Left: Representative H&E and Oil Red O staining of liver sections from mice fed an HFD for 16 weeks (n=6). Scale bars: 100 μm. Right: *Gpr75* mRNA expression in livers of HFD-fed mice measured by TaqMan probe and SYBR Green–based qRT-PCR (n=6). Bottom: GPR75 protein levels in livers of HFD-fed mice (n=4 biological replicates/group). (D) Light: H&E and Oil red O staining of liver sections from mice fed a CDAHFD for 6 weeks (n=6). Scale bars: 100 μm. Right: *Gpr75* mRNA expression in livers of CDAHFD-fed mice measured by Taqman probe and SYBR Green–based qRT-PCR (n=6). Bottom: GPR75 protein levels in livers of CDAHFD-fed mice (n=4 biological replicates/group). (E, F) Immunofluorescence staining of GPR75 in livers of mice fed an HFD (E) or CDAHFD (F). Scale bar: 50 μm (n=6). For all panels: *p<0.05, **p<0.01, ***p<0.001, ****p<0.0001, ns, no statistical significance. Data are presented as mean ± SEM; 2-tailed unpaired Student *t* test. Abbreviations: CDAHFD, choline-deficient, L-amino acid-defined high-fat diet; GPR75, G-protein–coupled receptor 75; HFD, high-fat diet; MASH, metabolic dysfunction–associated steatohepatitis; ND, normal diet.

### Liver *Gpr75* depletion alleviates MASH progression

To determine whether hepatic GPR75 upregulation mediates MASH progression, we generated mice with adeno-associated virus (AAV8)-mediated knockdown of hepatic *Gpr75* and fed them a CDAHFD diet (Figure 2A). Knockdown efficiency was confirmed at both mRNA and protein levels (Supplemental Figures S1C, D, http://links.lww.com/HEP/K371). *Gpr75* knockdown significantly reduced the liver/body weight ratio without affecting overall body weight (Figure 2B, Supplemental Figure S1E, http://links.lww.com/HEP/K371). Histological analysis revealed attenuated hepatic lipid accumulation and ballooning degeneration in hepatic *Gpr75* knockdown mice (Figure 2C), along with decreased levels of hepatic triglyceride (TG) triacylglycerol, total cholesterol, and nonesterified fatty acids (Figure 2D). We then examined the mRNA expression of genes involved in lipid metabolism to confirm which segment is impacted. Reduced hepatic GPR75 expression downregulated genes involved in fatty acid and TG synthesis but not those related to fatty acid β-oxidation or uptake (Figure 2E). These results implied that GPR75 may influence hepatic fatty acid synthesis.

**FIGURE 2:**
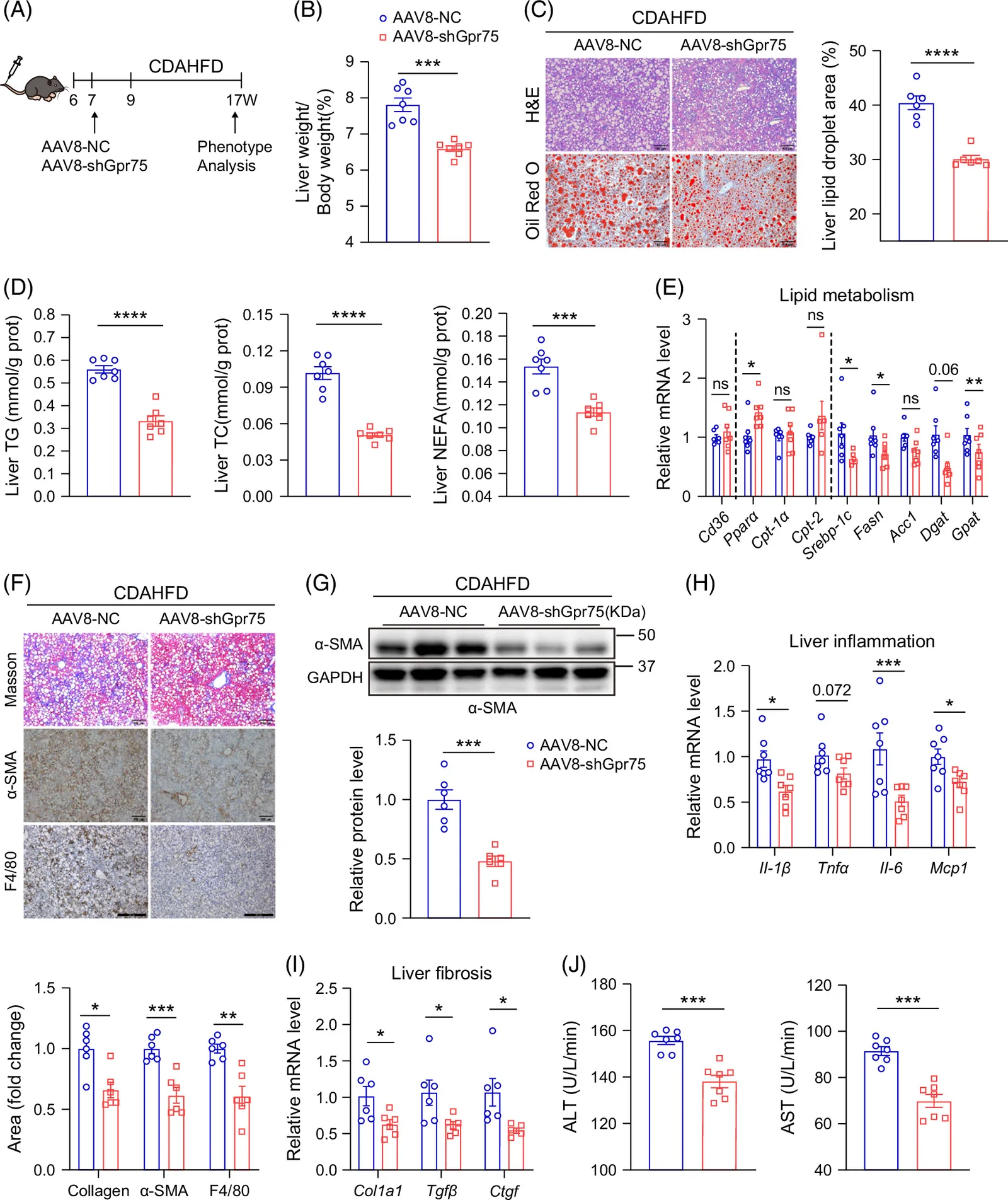
Liver *Gpr75* depletion alleviates MASH progression. (A) Schematic of *Gpr75* knockdown in the mouse liver to investigate its role in MASH. (B) Liver/body weight ratio in control and sh*Gpr75* mice (n=7). (C) Representative H&E and Oil Red O staining of liver sections. Scale bars: 100 μm (n=6). (D) Hepatic triglyceride, total cholesterol, and free fatty acid (FFA) levels (n=7). (E) qRT-PCR analysis of genes related to lipid uptake, fatty acid β-oxidation, and fatty acid synthesis in liver tissue (n=7). (F) Masson staining and IHC analysis of liver fibrosis. Scale bars: 100 and 200 μm (n=6). (G) Relative protein levels of α-SMA. GAPDH was used as a loading control (n=6 biological replicates / group). (H, I) mRNA expression of genes associated with (H) liver inflammation (n=6) and (I) fibrosis (n=7). (J) Serum ALT and AST levels (n=7). For all panels: *p<0.05, **p<0.01, ***p<0.001, ****p<0.0001, ns, no statistical significance. Data are presented as mean ± SEM; 2-tailed unpaired Student *t* test. Abbreviations: AAV8, adeno-associated virus 8; *Acc1*, Acetyl-CoA carboxylase 1; *Cd36*, Cluster of differentiation 36; COL1A1, collagen 1a1; *Cpt-1α*, Carnitine palmitoyltransferase 1 α; *Cpt-2*, Carnitine palmitoyltransferase 2; *Ctgf*, Connective tissue growth factor; *Dgat*, Diacylglycerol acyltransferase; *Fasn*, Fatty acid synthase; *Gpat*, Glycerol-3-phosphate acyltransferase; *Mcp1*, *Tgfβ*, Transforming growth factor β; NEFA, nonesterified fatty acids; *Pparα*, peroxisome proliferator-activated receptor α; *Scd1*, Stearoyl-CoA desaturase 1; *Srebp-1c*, sterol regulatory element-binding protein 1c; TC, total cholesterol; TG, triglyceride; *Tnfα*, tumor necrosis factor α; α-SMA, alpha-smooth muscle actin.

Given the central role of lipotoxicity, hepatocellular injury, inflammation, and fibrosis in MASH progression, we next evaluated the impact of *Gpr75* knockdown on these pathological features. Histological staining of Masson, picrosirius red and α-SMA, a marker protein of HSCs activation, suggested that *Gpr75* knockdown decreased the hepatic collagen deposition area. In addition, the expression of proinflammatory M1 macrophage markers (CD11b and F4/80) was significantly reduced (Figure 2F, Supplemental Figures S1F, G, http://links.lww.com/HEP/K371). The protein level of α-SMA was also markedly decreased, as confirmed by both western blot and immunofluorescence staining (Figure 2G, Supplemental Figure S1H, http://links.lww.com/HEP/K371). Supporting these findings, q-PCR analysis revealed downregulated mRNA expression of key fibrotic (*Col1a1, Tgfβ, Ctgf*) and inflammatory (*Tnfα, Il1β, Il6, Mcp1*) genes in the liver of *Gpr75*-knockdown mice (Figures 2H, I). The consistent protective effect against MASH progression was further evidenced by reduced serum ALT and AST levels (Figure 2J). Taken together, these data revealed that decreased expression of *Gpr75* protected against HFD-induced MASH and hepatic steatosis.

### Hepatocyte-specific *Gpr75* deficiency ameliorates hepatic steatosis and tissue damage

To delineate the cellular localization of upregulated GPR75 in MASH livers, we performed IF staining. The results revealed that GPR75 was primarily increased in Albumin^+^ hepatocytes (Figure 3A). Moreover, GPR75 levels were increased in mice primary hepatocytes and HepG2 cells upon palmitic acid (PA) treatment (Figure 3B). Therefore, hepatocyte-specific *Gpr75*-deficient mice were generated (*Gpr75* ^*hko*^ genotype: *Gpr75* ^*fl/fl*^ *TBG* ^*Cre*^) and subjected to a CDAHFD feeding for 8 weeks. Successful deletion of GPR75 was confirmed by IF staining of liver tissue (Figure 3C). *Gpr75* ^*hko*^ mice liver/body weight ratio was less (Figure 3D) and exhibited milder hepatic steatosis (H&E, Oil Red O, and Masson staining) than *Gpr75* ^*fl/fl*^ mice (Figure 3E). This protective phenotype was further supported by lower serum ALT and AST levels, decreased hepatic TG and nonesterified fatty acid content, and downregulated expression of lipid metabolism genes (Figure 3F, G). In the meantime, compared to *Gpr75* ^*fl/fl*^ controls, *Gpr75* ^*hko*^ mice livers showed reduced expression of α-SMA and extracellular matrix markers, indicating attenuated fibrosis (Figure 3H), alongside reduced macrophage infiltration and lower levels of inflammatory cytokines (*Il-1β, Il-6, Tnfα*) (Figures 3I, J).

**FIGURE 3:**
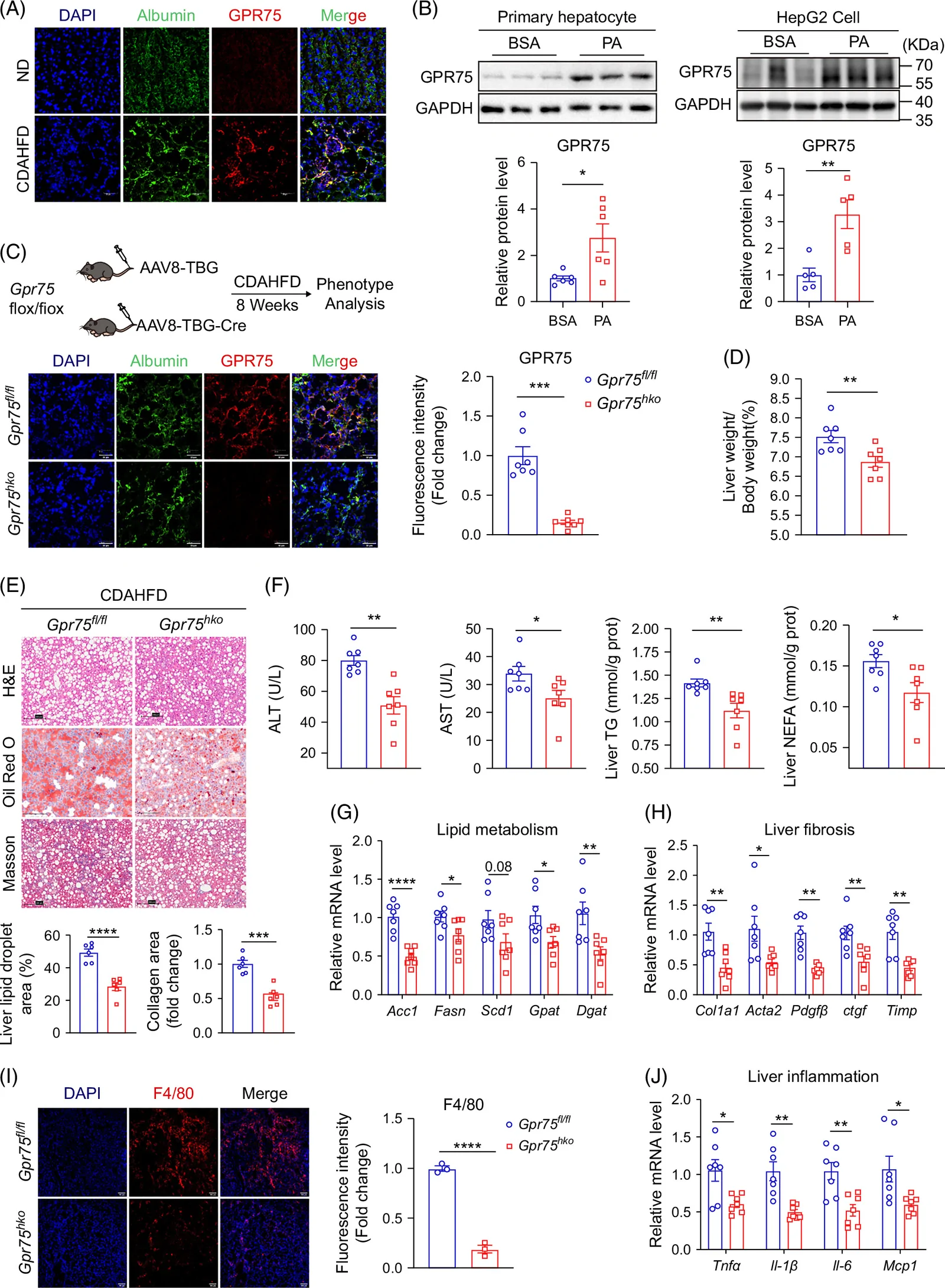
Hepatocyte-specific *Gpr75* deficiency ameliorates hepatic steatosis and tissue damage. (A) Immunofluorescence staining of GPR75 (red) and Albumin (green) in liver sections from mice fed a CDAHFD for 8 weeks. Scale bar: 50 μm (n=5). (B) Relative protein levels of GPR75 in primary hepatocytes and HepG2 cells, as determined by western blot (n=5–6 biological replicates/group). (C) Top: Schematic of hepatocyte-specific Gpr75 knockout mice under MASH diet feeding. Bottom: Immunofluorescence analysis of GPR75 in *Gpr75* ^*fl/fl*^ *and Gpr75* ^*hko*^ mice. Scale bar: 50 μm (n=7). (D) Liver/body weight ratio of *Gpr75* ^*fl/fl*^ and *Gpr75* ^*hko*^ mice (n=7). (E) Representative H&E, Masson trichrome, and Oil Red O staining of liver sections. Scale bars: 200 μm (n=6). (F) Serum ALT and AST activities, and TG and NEFA levels (n=7). (G) mRNA expression of lipid metabolism–related genes in liver tissue (n=7). (H) qRT–PCR analysis of fibrosis-associated gene expression (n=7). (I) Immunofluorescence staining of F4/80. Scale bar: 100 μm (n=3). (J) Relative mRNA expression of inflammatory genes (n=7). For all panels: *p<0.05, **p<0.01, ***p<0.001, ****p<0.0001, ns, no statistical significance. Data are presented as mean ± SEM; 2-tailed unpaired Student *t* test. Abbreviations: Acta2, actin alpha 2, smooth muscle; NEFA, nonesterified fatty acids; PA, palmitic acid; *Pdgfβ, platelet-derived growth factor subunit β*; TC, total cholesterol; TG, triglyceride; *Timp*, tissue inhibitor of metalloproteinases.

To confirm if hepatocyte-specific *Gpr75* ablation improves systemic metabolism, we subjected mice to a 12-week HFD regimen (Supplemental Figures S2A–D, http://links.lww.com/HEP/K371). While food intake was comparable (Supplemental Figure S2F, http://links.lww.com/HEP/K371), *Gpr75* ^*hko*^ mice gained less body weight than *Gpr75* ^*fl/fl*^ controls (Supplemental Figure S2E, http://links.lww.com/HEP/K371). Assessment of glucose homeostasis revealed that *Gpr75* ^*hko*^ mice had lower fasting blood glucose and enhanced insulin sensitivity (Supplemental Figures S2G–I, http://links.lww.com/HEP/K371), underscoring a key metabolic improvement. Hepatocyte-specific *Gpr75* depletion also ameliorated hepatic steatosis and reduced TG content (Supplemental Figures S2J–L, http://links.lww.com/HEP/K371). Concordantly, plasma levels of ALT, TG, and total cholesterol were significantly lower in *Gpr75* ^*hko*^ mice (Supplemental Figures S2M, N, http://links.lww.com/HEP/K371). Similar outcomes were observed in the expression of genes related to fatty acid synthesis, particularly a dramatic elevation in nuclear SREBP-1c levels. (Supplemental Figures S2O–Q, http://links.lww.com/HEP/K371). Interestingly, suspected TG, diacylglycerol, and cholesteryl ester were significantly reduced in the livers of *Gpr75* ^*hko*^ mice based on mass-to-nucleus ratio calculations using mass spectrometry imaging (Supplemental Figure S2R, http://links.lww.com/HEP/K371).

Collectively, these results demonstrate that hepatocyte-specific deletion of *Gpr75* ameliorates key features of established MASH in HFD-fed mice, including hepatic steatosis, fibrosis, inflammation, and injury.

### Hepatocyte-specific overexpression of *Gpr75* aggravates diet-induced MASH and fibrosis

Then, to determine if hepatic *Gpr75* overexpression exacerbates MASH, we performed AAV8-mediated, hepatocytes-specific *Gpr75* overexpression in mice (Figure 4A, Supplemental Figure S3A, http://links.lww.com/HEP/K371). Under a 6-week CDAHFD challenge, elevated *Gpr75* levels increased the liver/body weight ratio and aggravated hepatic steatosis, fibrosis, and macrophage infiltration compared to controls (Figures 4B, C, G, Supplemental Figures S3B, C, http://links.lww.com/HEP/K371). These changes were accompanied by increased serum ALT and AST activities (Figure 4D), higher hepatic TG, total cholesterol, and nonesterified fatty acid levels (Figure 4E). Quantitative PCR analysis revealed upregulation of genes involved in de novo lipogenesis (Figure 4F), fibrosis (Figures 4H, I), and inflammation (Figure 4J) in hepatocyte-specific *Gpr75*-OE mice, indicating enhanced liver damage and inflammatory response.

**FIGURE 4:**
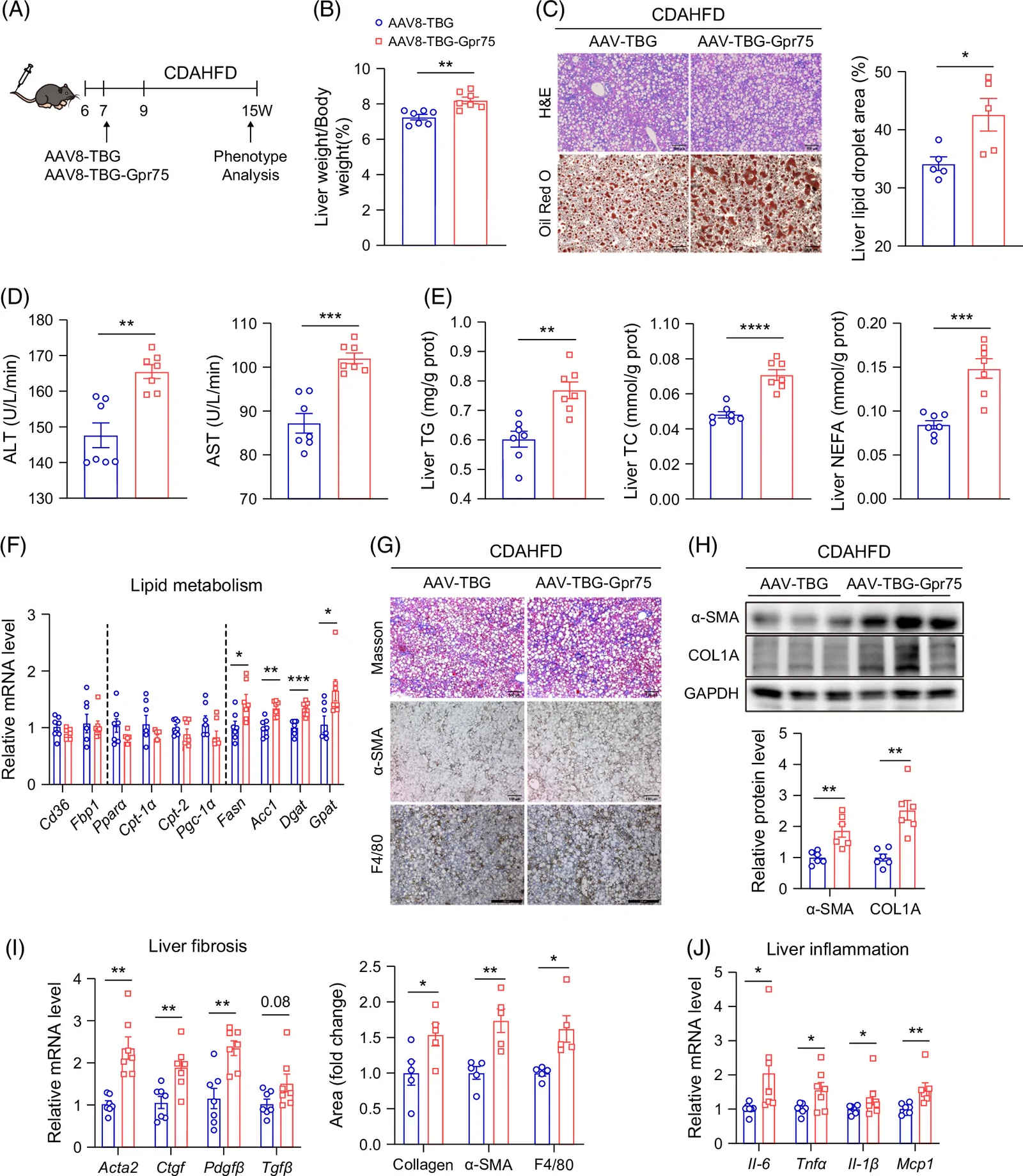
Hepatocyte-specific overexpression of *Gpr75* aggravates diet-induced MASH and fibrosis. (A) Experimental timeline for inducing MASH via CDAHFD feeding in hepatocyte-specific Gpr75-overexpressing mice. (B) Liver/body weight ratio (n=8). (C) Representative H&E and Oil Red O staining of liver sections. Scale bar: 200 μm (n=5). (D) Serum ALT and AST levels (n=8). (E) Hepatic TG, TC, and NEFA content (n=8). (F) mRNA expression of genes involved in FA metabolism (n=7). (G) Liver fibrosis assessment by Masson staining and IHC. Scale bar: 200 μm (n=5). (H) Relative protein expression of α-SMA and COL1A1, normalized to GAPDH (n=6 biological replicates/group). (I) mRNA expression of fibrosis-related genes (n=7). (J) mRNA expression of inflammation-associated genes (n=7). For all panels: *p<0.05, **p<0.01, ***p<0.001, ****p<0.0001, ns, no statistical significance. Data are presented as mean ± SEM; 2-tailed unpaired Student *t* test. Abbreviations: FA, fatty acid; NEFA, nonesterified fatty acid; TC, total cholesterol; TG, triglyceride.

We next examined the impact of *Gpr75* overexpression in an HFD-induced mouse model (Supplemental Figure S3D, http://links.lww.com/HEP/K371). Starting at the eighth week, *Gpr75-*OE mice showed significantly increased weight gain without changes in food intake, alongside impaired glucose homeostasis (Supplemental Figures S3E–H, http://links.lww.com/HEP/K371). Overexpression of *Gpr75* promoted severe hepatic steatosis and injury, evidenced by histology, increased serum AST, and elevated hepatic/serum lipid levels (Supplemental Figures S3I, J, L, and M, http://links.lww.com/HEP/K371). This phenotype was associated with enhanced expression of lipogenic genes and increased hepatic free fatty acid (Supplemental Figure S3K, http://links.lww.com/HEP/K371). Based on protein level and IF analyses, GPR75 likely upregulates nuclear SREBP-1c to promote lipid accumulation by inhibiting PKA phosphorylation (Supplemental Figures S3N, O, http://links.lww.com/HEP/K371).

In summary, contrasting the phenotype of *Gpr75* ^*hko*^ mice, hepatocyte-specific *Gpr75* overexpression exacerbates diet-induced steatosis, inflammation, and metabolic dysfunction, establishing GPR75 as a critical promoter of MASLD/MASH progression.

### GPR75 interacts with GNAI2 to regulate fatty acid synthesis

To investigate whether GPR75, as a G-protein–coupled receptor, promotes MASH progression through G-protein coupling, we performed a proteomic analysis of livers from CDAHFD-fed control and hepatic *Gpr75*-knockdown mice. The proteomic data have been deposited in the ProteomeXchange database under accession number PXD074927. Gene ontology enrichment revealed alterations in pathways involving catalytic activity, GTP binding, and peroxisome function (Figure 5A). Gene Set Enrichment Analysis further linked GPR75 to fatty acid metabolism, GTPase activity, and NF-kappa B signaling (Supplemental Figure S4A, http://links.lww.com/HEP/K371). More importantly, volcano plot analysis identified a significant decrease in the inhibitory G-protein subunit Gαi2 (encoded by *Gnai2*) upon *Gpr75* knockdown (Figure 5B). Previous studies have indicated that deletion of hepatic *Gnai2* in mice fed a methionine and choline-deficient diet reduces steatohepatitis, suppresses inflammatory markers, and enhances lipophagy .^23^ It was also shown that the transcriptional expression of *Gnai2* was upregulated by NF-κB in a TLR4-TRAF6–dependent manner, showing a positive feedback loop between GNAI2 and NF-κB that fosters a chronic inflammatory state in NASH.^23^ Furthermore, both mRNA and protein expression of GNAI2 were reduced by *Gpr75* knockdown and *Gpr75* Knockout (Supplemental Figures S4B–D, http://links.lww.com/HEP/K371) and elevated by its overexpression (Supplemental Figures S4E, F, http://links.lww.com/HEP/K371). To determine whether GNAI2 interacts with GPR75, Co-IP assay was performed in PA-treated HepG2 cells. As expected, the interaction between GNAI2 and GPR75 was verified (Figure 5C), which was further supported by their strong colocalization in the livers of MASH mice (Figure 5D).

**FIGURE 5:**
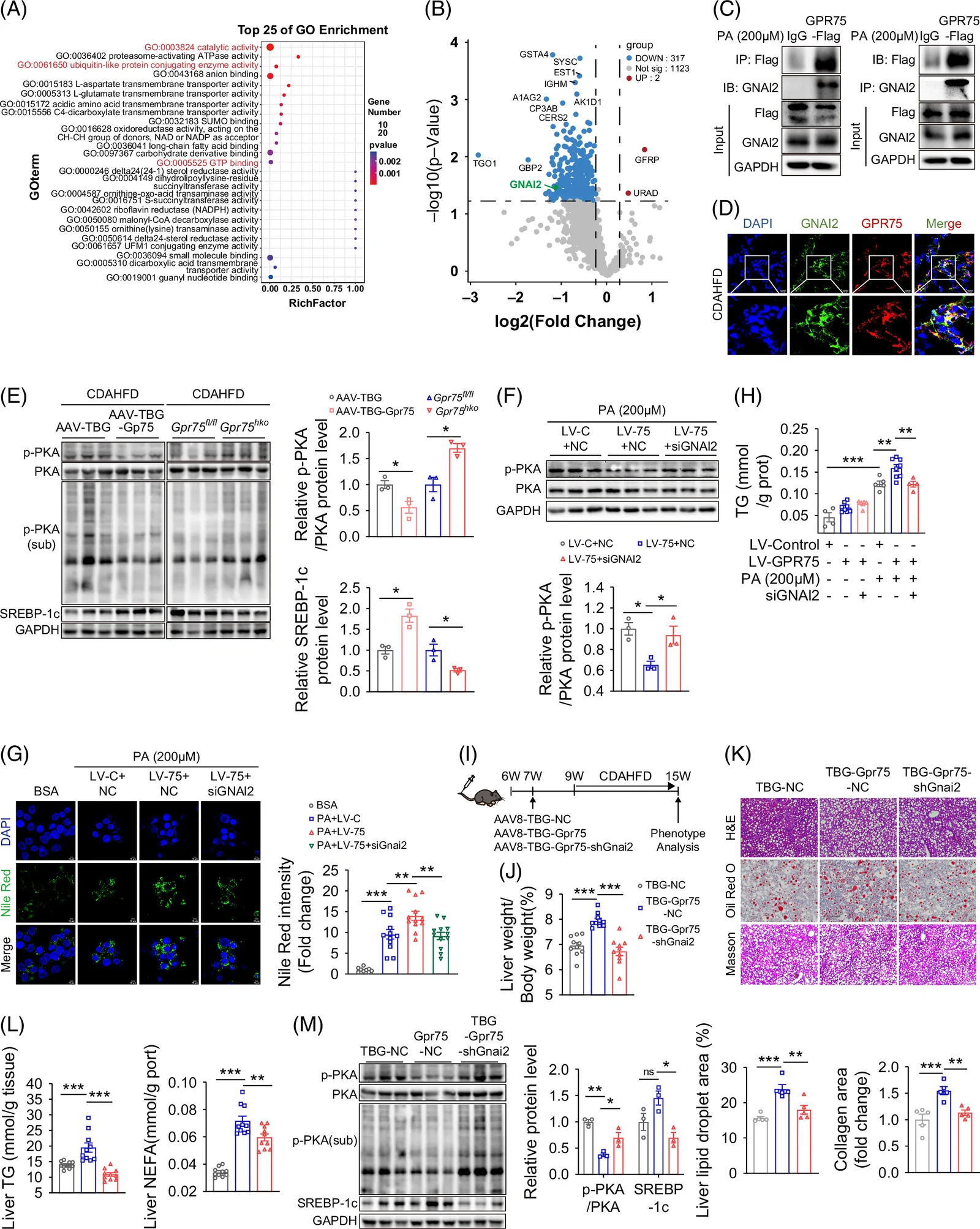
GPR75 interacts with GNAI2 to regulate fatty acid synthesis. GO enrichment analysis (A) and volcano plot (B) displaying significantly enriched signaling pathways and differentially expressed proteins. (C) Immunoprecipitation was performed using anti-Flag and GNAI2 antibodies in HepG2 cells (n=3 biological replicates/group). (D) IF staining showing colocalization of GPR75 (green) and GNAI2 (red) in liver sections (n=3). (E) Immunoblotting analysis of protein levels in livers from control, Gpr75-OE, *Gpr75* ^*fl/fl*^, and *Gpr75* ^*hko*^ mice after CDAHFD feeding (n=3 biological replicates/group). (F–H) Analyses in HepG2 cells transduced with LV-C (control), LV-GPR75, or LV-GPR75-siGNAI2 lentiviral vectors. (F) Protein levels of p-PKA and total PKA (n=3 biological replicates/group). (G) Lipid accumulation assessed by Nile Red staining. (H) Cellular TG content. (I) Experimental timeline for AAV8-TBG, AAV8-TBG-Gpr75 and AAV8-TBG–Gpr75 + AAV8-TBG–shGnai2 co-infected mice fed CDAHFD for 8 weeks. (J) Liver/body weight ratio (n=10). (K) Representative H&E, Oil Red O, and Masson trichrome staining of liver sections. Scale bar: 200 μm (n=5). (L) Hepatic TG and NEFA levels (n=10). (M) Western blot analysis of p-PKA, PKA substrate phosphorylation, total PKA, and SREBP-1c (n=3 biological replicates/group). For all panels: *p<0.05, **p<0.01, ***p<0.001, ****p<0.0001, ns, no statistical significance. Data are presented as mean ± SEM; 2-tailed unpaired Student *t* test for (E); 1-way ANOVA analysis for (F–H) and (J and M). Abbreviations: GNAI2, Guanine nucleotide-binding protein G(i) subunit alpha-2; GO, gene ontology; PKA, Protein Kinase A; SREBP-1c, Sterol Regulatory Element-Binding Protein 1c; TG, triglyceride.

Most GPCRs signal through one or more G proteins (eg, Gαi, Gαs, Gαq) and act on downstream effectors like adenylyl cyclases (ACs), phospholipase C, ion channels, and cGMP phosphodiesterase.^24,25^ AC catalyzes the synthesis of the second messenger cAMP, which in turn binds to PKA regulatory subunits (R), resulting in the release of active PKAc subunits.^24,25^ We hypothesized that the recruitment of GNAI2 plays a pivotal role in the pathogenic impact of GPR75 on exacerbating the progression of MASH. Specifically, it inhibits AC activity rather than being inhibited by AC. Overexpression of *Gpr75* suppressed PKA activity, thereby modulating the intracellular signaling molecules via GNAI2 and promoting the expression of SREBP-1c. Otherwise, *Gpr75* knockout released the inhibition on GNAI2 and enhanced the PKA phosphorylation activity, which in turn decreased SREBP-1c levels (Figure 5E). Furthermore, HepG2 cells infected with sh*GPR75* lentivirus exhibited decreased levels of SREBP-1c, TGs, and IL-1β, while increasing PKA phosphorylation (Supplemental Figures S5A–C, http://links.lww.com/HEP/K371). SREBPs (mainly SREBP1c and SREBP2) are critical nuclear transcriptional factors that regulate the DNL, cholesterol metabolism, and inflammation signaling pathways in hepatocytes involved in MASLD formation. More importantly, multiple evidence suggest that PKA phosphorylates SREBP-1c, thereby attenuating their transcriptional activity or regulating the maturation of precursor SREBP-1c, and identifies PKA as a potential activator for reducing lipid synthesis.^26^ The nuclear translocation of SREBP-1c and both the precursor (P) and the mature (N) form of SREBP-1c was markedly decreased by GPR75 depletion (Supplemental Figure S5D, http://links.lww.com/HEP/K371). By contrast, overexpression of *GPR75* in hepatocytes effectively inhibited the phosphorylation of PKA, while increasing SREBP-1c protein level and TG content. These effects were diminished by *GNAI2* silencing in a lentiviral transduction-generated stable cell line (Figure 5F-H, Supplemental Figures S5E, F, http://links.lww.com/HEP/K371).

To validate the functional relationship between GPR75 and GNAI2 in *vivo*, we co-administered AAV-TBG-Gpr75 or AAV-TBG-Gpr75-shGnai2 to mice (Figure 5I, Supplemental Figure S5G, http://links.lww.com/HEP/K371). Knockdown of *Gnai2* mitigated the hepatomegaly and hepatic steatosis induced by *Gpr75* overexpression, as evidenced by reduced liver weight, improved histology (H&E and Oil Red O staining), and lower TG levels (Figures 5J–L). Additionally, the upregulation of DNL genes by GPR75 was also inhibited (Supplemental Figure S5H, http://links.lww.com/HEP/K371). To demonstrate the contribution of GNAI2 to the GPR75-mediated regulation of liver damage, inflammation, and fibrosis progression, we measured serum AST levels and the protein levels of α-SMA and F4/80. The results showed that *Gnai2* silencing significantly attenuated the severe MASH progression induced by *Gpr75* overexpression (Supplemental Figure S5I–K, http://links.lww.com/HEP/K371). As expected, *Gpr75* overexpressing inhibited PKA activity, leading to a decrease in the phosphorylation of PKA substrates, while this regulatory effect of GPR75 on PKA was abolished following *Gnai2* knockdown (Figure 5M). Overall, our data strongly suggest that suppression of *Gnai2* significantly alleviates *Gpr75*-overexpression-induced liver inflammation and fibrosis.

### VPS35 maintains GPR75 expression by recycling GPR75 to the hepatocyte surface

To explain the discordance between the upregulation of GPR75 protein levels and its unchanged mRNA expression in MASH, we investigated the degradation of GPR75 protein. The degradation rate of GPR75 slowed down after PA exposure (Figure 6A). Among the proteins that interact with GPR75, as identified by immunoprecipitation and mass spectrometry, we found several candidates implicated in protein degradation pathways, including UBE2N (Figure 6B, Supplemental Figure S6A, http://links.lww.com/HEP/K371). However, there was no direct interaction between UBE2N and GPR75, and almost no changes at the ubiquitination level of GPR75 when treated with an UBE2N inhibitor, NSC697923 (Supplemental Figures S6B, C, http://links.lww.com/HEP/K371).

**FIGURE 6:**
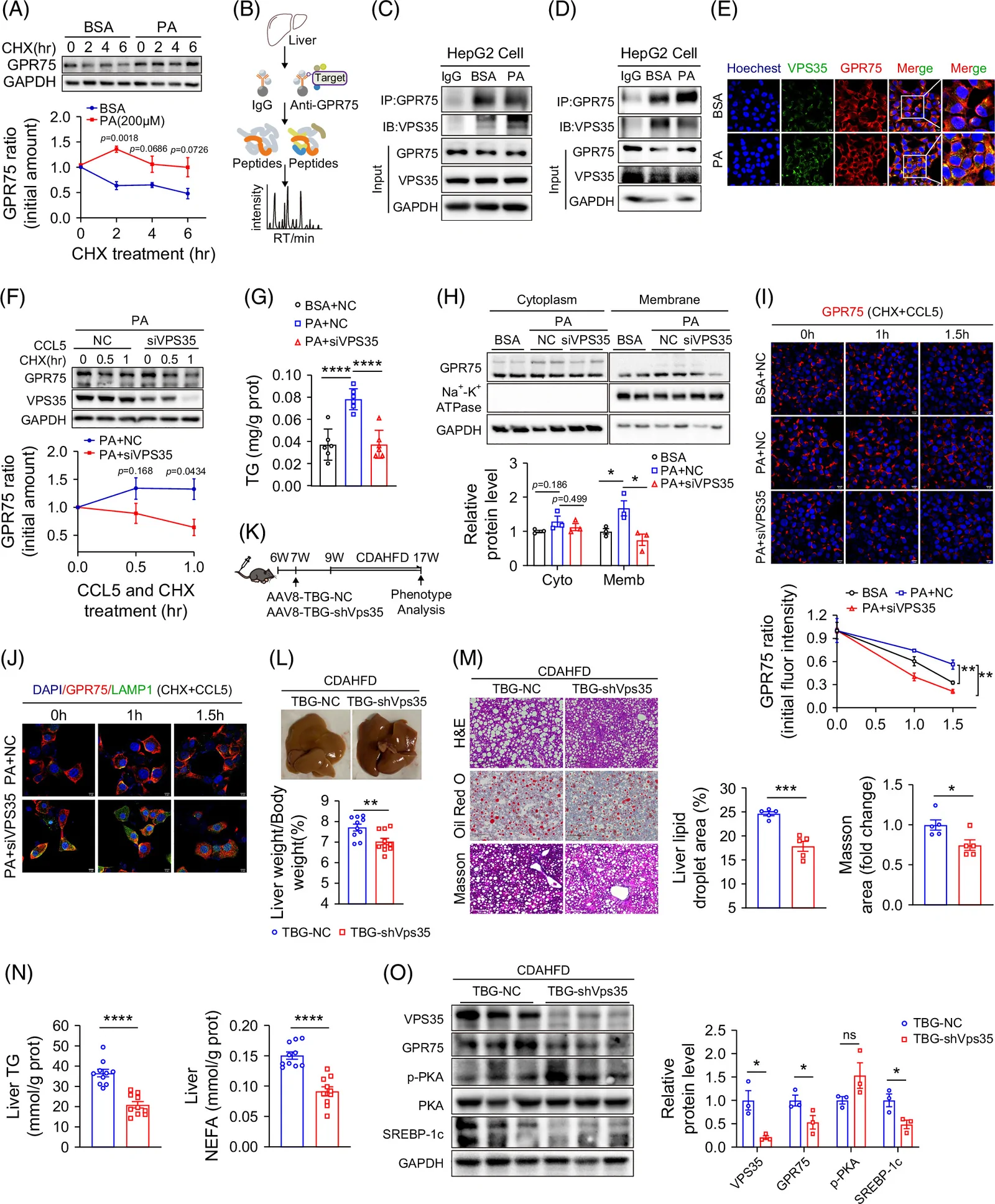
VPS35 maintains GPR75 expression by recycling GPR75 to the hepatocyte surface. (A) Relative protein expressions of GPR75 in HepG2 cells stimulated with or without PA, followed by cycloheximide (CHX, 100 μg/mL) for 0, 2, 4, and 6 hours (n=3 biological replicates/group). (B) Schematic of IP-MS assays. (C, D) co-IPs analysis of GPR75 and VPS35 interaction in HepG2 cells (n=2 biological replicates/group). (E) Fluorescence imaging showing colocalization of GPR75 and VPS35 in AML12 cells. (F) Relative protein levels of GPR75 in HepG2 cells (n=3 biological replicates/group). (G) TG content in HepG2 cells. (H) Immunoblot analysis of membrane and cytoplasmic proteins from HepG2 cells. (I) Confocal microscopy images of GPR75 in AML12 cells. Scale bar: 15 μm. (J) Colocalization analysis of GPR75 (red) and LAMP1 (green). Scale bar: 15 μm. (K) Experimental design for hepatocyte-specific *Vps35* knockdown in mice fed a CDAHFD for 8 weeks. (L) Liver/body weight ratio in mice (n=10). (M) Representative H&E, Oil Red O, and Masson staining of liver sections. Scale bar: 200 μm (n=5). (N) hepatic TG and NEFA levels (n=10). (O) Relative protein expressions of VPS35, GPR75, p-PKA, PKA, and SREBP-1c in liver tissues (n=3 biological replicates / group). For all panels: *p<0.05, **p<0.01, ***p<0.001, ****p<0.0001, ns, no statistical significance. Data are presented as mean ± SEM; 1-way ANOVA analysis for G–I; 2-tailed unpaired Student *t* test for A, F, and L–O. Abbreviations: CCL5, C-C Motif chemokine ligand 5; LAMP1, lysosome-associated membrane glycoprotein 1; PA, palmitic acid; TG, triglyceride; VPS35, vacuolar protein sorting-associated protein 35.

Gene ontology biological process analysis revealed an enrichment of pathways related to vesicle-mediated transport (Supplemental Figure S6D, http://links.lww.com/HEP/K371). Among these, VPS35—a core component of the retromer complex, which mediates interactions with sorting receptors destined for recycling^27–29^—was identified. Interestingly, VPS35 showed significant intercellular colocalization with GPR75 in both HepG2 cells (Figure 6E) and CDAHFD-induced mouse livers (Supplemental Figure S6E, http://links.lww.com/HEP/K371). Co-IP assays also confirmed that VPS35 and GPR75 could bind each other, indicating that VPS35 may promote sorting of GPR75 for delivery back to the membrane, rather than to the lysosome or proteasome (Figures 6C, D).

*VPS35* silencing accelerated GPR75 degradation regardless of CCL5 stimulation (Figure 6F, Supplemental Figure S6F, http://links.lww.com/HEP/K371) and dramatically attenuated PA-induced lipid accumulation in hepatocytes (Figure 6G), supporting its essential role in stabilizing GPR75. More importantly, immunoblotting and immunofluorescence analyses confirmed that upregulation of GPR75 occurred predominantly at the cell membrane, with no significant increase in the cytoplasm (Figures 6H, I). This finding further demonstrates that VPS35 promotes GPR75 recycling to the cell surface, thereby enhancing its membrane localization and reducing its degradation. In line with these findings, *siVPS35* enhanced GPR75 colocalization with the lysosomal marker LAMP1 (Figure 6J). These results indicate that VPS35 sorts and recycles GPR75 to the plasma membrane, thereby maintaining its high expression and functional activity.

Consistent with its pathogenic role, *VPS35* was upregulated in the liver of both diet-induced mice and patients with MASH (Supplemental Figures S6G, H, http://links.lww.com/HEP/K371). To investigate its functional significance, we knocked down hepatic *Vps35* in CDAHFD-induced mice using AAV8-TBG-shVps35 (Figure 6K, Supplemental Figure S6I, http://links.lww.com/HEP/K371). Depletion of hepatic *Vps35* ameliorated the MASH phenotype, as indicated by liver weight/body weight ratio, lower TG and free fatty acid levels, suppressed expression of lipogenic genes, and improved histology (Figure 6L-N, Supplemental Figure S6J, http://links.lww.com/HEP/K371). Concurrently, *Vps35* knockdown blocked liver injury induced by CDAHFD, significantly decreased the accumulation of hepatic F4/80^+^ macrophages within the liver, reduced hepatic α-SMA-positive area, and ameliorated liver injury (Supplemental Figures S6K–M, http://links.lww.com/HEP/K371). The most importantly, the protein expression of GPR75 was strongly reduced in *Vps35* knockdown mice (Supplemental Figure S6N, http://links.lww.com/HEP/K371), without changes at the transcriptional level (Supplemental Figure S6I, http://links.lww.com/HEP/K371). Regarding the downstream pathway of GPR75, *Vps35* knockdown activated PKA phosphorylation, similar to the effects of hepatic *Gpr75* knockdown (Figure 6O). Collectively, these data suggest that *Vps35* knockdown reduces the transport of GPR75 back to the hepatocyte membrane and that VPS35 expression is positively correlated with MASH severity.

### Hepatic GPR75 is a potential target for screening MASH therapy compounds

Based on the data above, we screened compounds with high binding affinity to GPR75 using computer-aided molecular docking (AutoDock and MolProphet V1.0) followed by functional validation in a BODIPY staining assay. Among these compounds, X201415 (CAS 477252-30-5) was identified based on its potent, dose-dependent inhibition of lipid droplet accumulation in HepG2 cells (Figures 7A, B). We also conducted a cellular thermal shift assay to evaluate the binding between GPR75 and X201415. The result showed that X201415 increased the thermal stability of GPR75 (Figure 7C). Deletion of *GPR75* abolished the protective effect of X201415, suggesting that X201415 acts against lipid accumulation in hepatocytes via interaction with GPR75 (Figure 7D). To evaluate X201415 as a novel MASH therapeutic candidate, we assessed its pharmacological effect in CDAHFD-induced MASH mice (20 mg/kg, i.g., once per day). As expected, X201415 reduced body weight, alleviated hepatic steatosis in histology, and lowered liver TG (Figures 7E–H). At the same time, the protective effects of this compound against diet-induced hepatic lipid synthesis, inflammatory activation, and fibrosis were consistently observed (Figures 7I–K). Furthermore, compared to vehicle controls, the treatment group showed increased activation of the cAMP-PKA pathway in the liver and decreased SREBP-1c protein levels (Figure 7L). Taken together, these results indicate that X201415, by targeting GPR75, is effective in MASH therapy, highlighting the potential of hepatic GPR75 as a target for MASH therapy.

**FIGURE 7:**
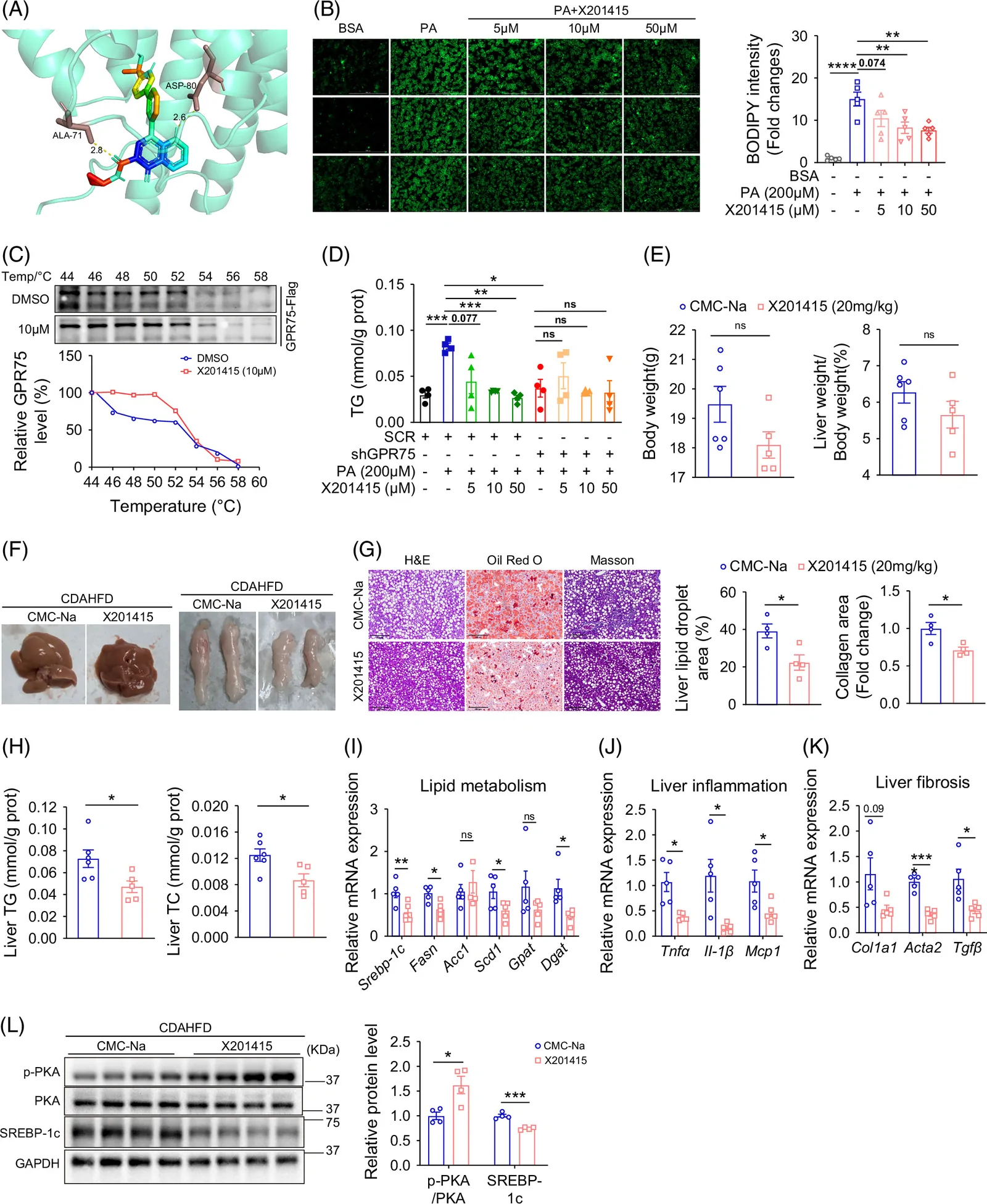
Hepatic GPR75 is a potential target for screening MASH therapy compounds. (A) Molecular docking model of compound X201415 bound to GPR75 (green). (B) Lipid droplet content assessed by BODIPY staining in HepG2 cells treated with BSA, PA, and X201415. (C) Cellular thermal shift assay (CETSA) followed by Western blot analysis evaluating the binding between X201415 and GPR75 (n=3 biological replicates/group). (D) Cellular TG content in HepG2 cells. (E) Body weight and liver/body weight ratio of mice fed a CDAHFD for 4 weeks and administered 20 mg/kg/d X201415 or vehicle (CMC-Na) via gavage during the final 2 weeks (n=6 for CMC-Na, n=5 for X201415). (F) Macroscopic appearance of livers and gonadal white adipose tissue (gWAT). (G) Representative H&E, Oil Red O, and Masson trichrome staining of liver sections. Scale bar: 200 μm (n=3). (H) Hepatic triglyceride (TG) and TC levels (n=5–6). (I–K) mRNA expression of lipogenesis-related genes (I), proinflammatory factors (J), and fibrosis-associated genes (K) (n=5). (L) Western blot analysis of p-PKA, total PKA, and SREBP-1c in livers of vehicle- and X201415-treated mice (n=4 biological replicates/group). For all panels: *p<0.05, **p<0.01, ***p<0.001, ****p<0.0001, ns, no statistical significance. Data are presented as mean ± SEM; one-way ANOVA analysis for (B and D); 2-tailed unpaired Student *t* test for E and G–L. Abbreviations: CMC-Na, Sodium carboxymethyl cellulose; SCR, scrambled control (carried by) virus; TC, total cholesterol.

## DISCUSSION

Preliminary studies revealed that GPR75 is predominantly expressed in cells surrounding retinal arterioles and various brain regions. Subsequent research has implicated GPR75 in cerebrovascular disease, colon cancer, hypertension, and obesity.^30–34^ While some studies have explored the link between GPR75 and MASLD, they have primarily focused on its impact on food intake under HFD conditions to normalize body weight and alleviate liver steatosis.^9,35^ Consistent with a minor hepatic role, analysis of extensive databases indicates that GPR75 is broadly expressed across most human tissues but is notably low in the liver. These databases, largely based on RNA-sequencing, suggest that *Gpr75* is not highly expressed at the RNA level in the liver, a finding corroborated by our quantitative PCR analysis. However, we observed an intriguing anomaly in the livers of MASH mice induced by an HFD, where GPR75 protein expression showed a marked increase. This unexpected finding piqued our interest, leading us to conduct functional verification experiments. Our results confirmed that GPR75 in the liver indeed plays a crucial role in mediating MASH progression.

Like other GPCRs, GPR75 has specific ligands. Ignatov et al^20^ reported that CCL5 increased IP_3_ and intracellular Ca^2+^ levels via a Gq protein–coupled pathway in CHO or HEK cells overexpressing mouse *Gpr75*. A recent study demonstrated that CCL5 stimulates insulin secretion in isolated islets via phospholipase C-activated Ca^2+^ influx in a GPR75-dependent manner; however, this study did not document direct binding or interaction between GPR75 and CCL5.^21^ Additionally, some data suggest that GPR75 may act as the receptor for 20-HETE, capable of binding 20-HETE with high affinity and activating a signaling cascade that cause many of the functional outcomes attributed to 20-HETE both in vitro and in vivo. In our study, CCL5 was used as an agonist for GPR75 to activate the downstream effects of GPR75 and observe the effects of the coupled G-protein.

Heterotrimeric G proteins, which are activated by ligand-bound GPCRs, are functionally determined by their Gα subunit. Upon activation, GDP is exchanged for GTP on the Gα subunit, triggering its dissociation from the Gβγ dimer; both components then regulate downstream effectors.^36,37^ Based on the characteristics of the Gα subunit, heterotrimeric G proteins are classified into 4 families: Gαs (stimulation), Gαi (inhibition), Gαq, and Gα12.^38^ The 3 isoforms of Gαi (Gαi1, Gαi2, Gαi3), encoded by the genes Gnai1, Gnai2, and Gnai3, respectively, act as inhibitory Gα subunits that suppress AC activity, resulting in decreased intracellular cAMP levels.^37^ In our study, we found a definite interaction between GPR75 and GNAI2, suggesting that GPR75 initiates downstream cascade reactions by coupling with Gαi2. Importantly, Gαi2 could play an essential role in MASH progression, with its expression significantly elevated in MASH patients’ liver tissues.^39^ Additionally, Gαi2 deficiency increases lipolytic activity in brown and white adipocytes in a cAMP-dependent manner, likely explaining increased energy expenditure and significantly reduced weight gain under high caloric conditions.^40^

Furthermore, we have confirmed the involvement of the downstream PKA signaling pathway of GNAI2 in regulating lipid synthesis. This is consistent with recent studies demonstrating that PKA, a mediator of glucagon/cAMP signaling during fasting, suppresses SREBP-1c by phosphorylating and inhibiting liver X receptor α (LXRα).^41,42^ Both in vitro and in vivo evidence have shown that PKA directly phosphorylates LXRα at Ser195/196 and Ser290/291 within its ligand-binding domain, thereby suppressing *Srebp-1c* mRNA expression and the synthesis of fatty acids and TGs in the liver.^41^ Additional evidence indicates that thyroid-stimulating hormone, via its receptor (TSHR), enhances hepatic SREBP-1c activity through the cAMP/PKA/PPARα pathway, which is associated with reduced AMPK activity, leading to hepatic TG accumulation.^43^ The cleavages release the N-terminal portions of the SREBPs, which translocate to the nucleus as transcription factors, enhancing the transcription of genes necessary for de novo lipogenesis, such as *Acc1*, *Fasn*, *Scd1*, and *Gpat*.^44^ These results are consistent with our findings.

Our results show a significant increase in hepatic GPR75 protein in mice fed a HFD or choline-deficient, high-fat diet (CDAHFD), suggesting that GPR75 is regulated at the post-transcriptional level in MASH. Moreover, our proteomic analysis revealed an enrichment of vesicle transport proteins, with VPS35 was identified as a key interacting partner. Data from our animal models, along with analysis of the GEO database, indicate that both mRNA and protein levels of VPS35 are elevated in MASLD livers. Although the specific function of VPS35 in MASLD remains poorly understood, its well-established role in regulating cell surface receptor trafficking in central nervous system disorders and cancers provides a crucial mechanistic context for interpreting our findings.^45^ GPCRs are synthesized in the rough endoplasmic reticulum, packaged into transport vesicles, and trafficked via the Golgi apparatus to the plasma membrane through anterograde transport. However, these receptors are not static; they frequently cycle internally (through retrograde transport) and return to the cell surface. During these cycles, GPCRs fulfill their biological roles when the vesicles carrying them are directed toward lysosomes or the proteasome for degradation.^46^ This trafficking pattern highlights the intricate regulation of GPCRs and the importance of precise sorting mechanisms. Our research demonstrates that GPR75 relies on VPS35 for its selective sorting and subsequent transportation back to the cell membrane. This process ensures sustained high expression levels of GPR75 in MASH mice, emphasizing the critical role of VPS35 in regulating receptor membrane availability.

In summary, our research has uncovered a significant increase in the expression of GPR75 protein within the liver during the MASH state, rather than at the mRNA level. This phenomenon is intricately linked to the transport functions of VPS35, which plays a crucial role in enhancing the stability of the GPR75 protein and facilitating its recycling to the cell surface in MASH mouse livers. Furthermore, silencing *Vps35* results in a substantial decline in GPR75 protein levels, which is accompanied by a reduction in PKA phosphorylation and subsequent suppression of SREBP-1c expression. Importantly, the depletion of hepatic *Gpr75* has been shown to ameliorate MASH in mice, suggesting that it may be a key factor in the disease process. The deficiency of hepatic *Gpr75* reduces de novo lipogenesis through the GNAI2-cAMP-PKA signaling pathway, highlighting its potential as a therapeutic target for MASH. Overall, these findings provide valuable insights into the molecular mechanisms underlying MASH and suggest new avenues for potential treatment strategies.

## Supplementary Material
