# Supplementary material for: Hepatic GPR75 exacerbates MASH through GNAI2-dependent signaling
Source: Hepatology. 2026 Feb 3;84(5):1469–85. doi: 10.1097/HEP.0000000000001706 (PMC13668042; doi:10.1097/HEP.0000000000001706)
Supplement: Supplementary file 1 [file hep-84-1469-s001.docx]

**HEP-25-2405-revision**

**Supplemental information**

**Hepatic GPR75 Exacerbates MASH through GNAI2-Dependent Signaling**

**Authors**

Xule Yang^1#^, Na Yang^2#^, Zhihao Cheng^1^, Rui Zhang^1^, Hui Fan^1^, Dongsheng Liu^1^, Jiye Aa^1^, Guangji Wang^1,3^*, Yuan Xie^1^*

**Supplemental Figures and Figure Legends**

**Supplemental Figure S1. Liver *Gpr75* depletion alleviates MASH progression. Related to Figure. 2.**

(A) The mRNA levels of hepatic *Gpr75* in HFD-induced mice were measured by qRT-PCR using primer sequence referenced from a study published in *Cell metabolism* ([doi.org/10.1016/j.cmet.2024.03.016](https://doi.org/10.1016/j.cmet.2024.03.016)).

(B) The protein levels of GPR75 in mouse brain and liver were detected using a GPR75-specific antibody, with a corresponding blocking peptide (n=3-6 biological replicates / group).

(C) Hepatic mRNA (n = 7) expression levels of GPR75 in mice fed a CDAHFD diet.

(D) The protein levels of GPR75 in mice fed with ND and CDAHFD diet (n=3 biological replicates / group).

(E) Body weight of control and sh*Gpr75* mice (n = 7).

(F) Sirius Red and CD11b staining of liver sections. Scale bar: 200 μm (n = 5).

(G, H) Representative immunofluorescence images of α-SMA (G) and F4/80 (H) staining in liver sections from control and sh*Gpr75* mice. Scale bar: 100 μm (n = 3).

For all panels: **p* < 0.05, ***p* < 0.01, ****p* < 0.001, ns, no statistical significance. Data are presented as mean ± SEM. Two-tailed unpaired Student’s t test.

**Supplemental Figure S2. Hepatocyte-specific *Gpr75* deficiency ameliorates hepatic steatosis and tissue damage.** **Related to Figure. 3.**

(A) Schematic of the loxP site insertion into the *Gpr75* gene for generating hepatocyte-specific knockout (HKO) mice via gene editing.

(B) Schematic diagram of the experimental design in HFD-induced mice.

(C) Hepatic mRNA expression of *Gpr75* in HFD-induced *Gpr75^fl/fl^* and *Gpr75^hko^* mice (n = 12).

(D) Protein levels of GPR75 in the liver or hepatocytes of HFD-induced *Gpr75^fl/fl^* and *Gpr75^hko^* mice (n=3 biological replicates / group).

(E, F) Body weight and food intake curve of mice (n = 12).

(G) Blood glucose levels (n = 12).

(H) Glucose tolerance test (GTT) and (I) insulin tolerance test (ITT) (n = 8).

(J) Representative images of liver (top) and liver/body weight ratio (n = 12).

(K) Representative liver sections stained with H&E and Oil Red O (n = 5).

(L) Hepatic TG, NEFA levels (n = 12).

(M) Serum ALT levels (n = 12).

(N) Serum TG, TC, FFA levels (n = 12).

(O) qRT-PCR analysis of mRNA expression of lipid synthesis-related genes (n = 12). (P) Relative protein levels of p-PKA, PKA and SREBP-1c in mouse livers (n = 3 biological replicates / group).

(Q) Immunofluorescence staining shows nuclear SREBP-1c in livers of *Gpr75* KO mice Scale bar: 15 μm (n = 3).

(R) DESI-MS imaging analysis of metabolites in livers of *Gpr75^fl/fl^* and *Gpr75^hko^* mice fed with HFD (n = 3).

For all panels: **p* < 0.05, ***p* < 0.01, ****p* < 0.001, *****p* < 0.0001, ns, no statistical significance. Data are presented as mean ± SEM. Two-tailed unpaired Student’s t test.

**Supplemental Figure S3. Hepatocyte-specific *Gpr75* deficiency ameliorates hepatic steatosis and tissue damage. Related to Figure. 4.**

(A) Relative hepatic *Gpr75* mRNA expression in control (AAV8-TBG) mice and *Gpr75*-OE (AAV8-TBG-Gpr75) mice induced with CDAHFD for 6 weeks (n =8).

(B, C) Representative immunofluorescence images of α-SMA (B) and F4/80 (C). Scale bar: 100 μm (n = 3).

(D) Schematic of experimental design for hepatocyte-specific *Gpr75* overexpression in HFD-induced mice (top). qRT-PCR analysis of *Gpr75* mRNA expression in livers of mice (bottom) (n = 8).

(E, F) Body weight and food intake curve of mice (n =8).

(G) Blood glucose levels (n = 8).

(H) Glucose tolerance test (GTT) (n = 8).

(I) H&E and Oil Red O staining of mouse liver sections. Scale bar: 200 μm (n = 5).

(J) Hepatic TG, NEFA, TC content (n = 8).

(K) mRNA expression of lipid synthesis-related genes (n = 7).

(L) Serum TG, TC and NEFA levels and (M) serum AST levels (n = 8).

(N) The protein levels of p-PKA, total PKA and SREBP-1c in the livers (n = 3 biological replicates / group).

(O) Immunofluorescence staining shows nuclear SREBP-1c in livers of *Gpr75*-OE mice. Scale bar: 15 μm (n = 3).

For all panels: **p* < 0.05, ***p* < 0.01, ****p* < 0.001, *****p* < 0.0001, ns, no statistical significance. Data are presented as mean ± SEM. Two-tailed unpaired Student’s t test.

**Extended Data Fig. 4: GPR75 affects GNAI2 expression in MASH mice.** **Related to Figure. 5.**

(A) Gene set enrichment analysis (GSEA) of enrichment pathways for significantly altered genes in *Gpr75* knockdown mice compared to controls (n = 6).

(B and C) Analysis of GNAI2 expression in livers of control and sh*Gpr75* mice: (B) The protein levels of GNAI2 *(n = 3 biological replicates / group)*; (C) Relative mRNA expression of *Gnai2* (n = 7).

(D) GNAI2 expression in livers of *Gpr75^fl/lf^* and *Gpr75^hko^*mice. Left: The protein levels of GNAI2 (n = 3 biological replicates / group); right: Relative mRNA expression of *Gnai2* (n = 12).

(E and F) Analysis of GNAI2 expression in livers of control and *Gpr75*-OE mice: (E) The protein levels of GNAI2 (n = 3 biological replicates / group). (F) Relative mRNA expression of *Gnai2* (n = 7).

For all panels: **p* < 0.05, ***p* < 0.01, ****p* < 0.001, *****p* < 0.0001, ns, no statistical significance. Data are presented as mean ± SEM. Two-tailed unpaired Student’s t test.

**Extended Data Fig. 5: GPR75 regulated the expression of SREBP-1c to affect the hepatocyte lipid metabolism.** **Related to Figure. 5.**

(A, B) The protein levels of Control and *GPR75* stable knockdown HepG2 cells treated with BSA or palmitic acid (PA, 200 μM) for 24 h (n = 3 biological replicates / group).

(C) Cellular TG levels of HepG2 cells.

(D) Immunoblot analysis of SREBP-1c protein levels in Cytoplasmic and nuclear fractions from HepG2 cells infected with scramble control (SCR) or sh*GPR75* lentivirus. (n = 1 biological replicate / group).

(E) Immunoblot analysis of SREBP-1c protein levels in Cytoplasmic and nuclear fractions from HepG2 cells infected with control lentivirus (LV-C) and GPR75-overexpression lentivirus ((LV-75) (n = 1 biological replicate / group).

(F) Quantification of cellular TG levels.

(G) Hepatic *Gpr75* and *Gnai2* mRNA expression in mouse livers (n = 7).

(H) Relative mRNA expression of hepatic fatty acid synthesis and TG formation-related genes (n = 7).

(I, J) Representative IF images of α-SMA (I) and F4/80 (J) in liver sections. Scale bar: 100 μm (n = 3).

(K) Serum AST levels (n = 10).

For all panels: **p* < 0.05, ***p* < 0.01, ****p* < 0.001, ns, no statistical significance. Data are presented as mean ± SEM. One-way ANOVA analysis.

**Extended Data Fig. 6: VPS35 maintains GPR75 expression by recycling GPR75 to hepatocyte surface. Related to Figure. 6.**

(A) IP-mass spectrometry analysis of GPR75-interacting proteins in livers of CDAHFD-induced mice.

(B) co-IP analysis of the interaction between GPR75 and UBE2N in HepG2 cells infected with LV-GPR75-3 x Flag lentivirus and treated with BSA or PA (n = 1 biological replicate / group).

(C) IP assays evaluating ubiquitination levels of GPR75 in HepG2 cells (n = 1 biological replicate / group).

(D) GO analysis showing the top 20 enriched pathways from IP-MS data.

(E) Representative immunofluorescence images showing colocalization of GPR75 and VPS35 in liver sections from CDAHFD-fed mice.

(F) Relative protein levels of GPR75 in HepG2 cells transfected with negative control (NC) or si*VPS35* stimulating with PA (n = 3 biological replicates / group).

(G) Proteomic analysis of VPS35 protein levels in mouse liver (n = 5).

(H) *VPS35* mRNA levels in human liver samples from patients with Simple Steatosis (SS), NASH, or healthy controls (GSE89063, GSE17470, GSE89632).

(I) The mRNA expression of *Vps35* and *Gpr75* in liver tissues (n = 6).

(J) Relative mRNA expression of genes related to FA and TG synthesis (n = 7).

(K) Serum AST and ALT levels (n =10).

(L-N) IF staining of α-SMA (L), F4/80 (M), and GPR75 (N) in livers of control and hepatocyte-specific *Vps35* knockdown mice (n = 3).

For all panels: **p* < 0.05, ***p* < 0.01, ****p* < 0.001, *****p* < 0.0001, ns, no statistical significance. Data are presented as mean ± SEM. Two-tailed unpaired Student’s t test for (G-K); one-way ANOVA analysis for (F) and (H).

**SUPPLEMENTARY METHODS**

**Mouse experiments**

For experiments, 6- to 8-week-old male mice were used and littermate mice served as controls across all genotypes. *Gpr75* flox/flox mice (C57BL/6Smoc-*Gpr75^em1(flox)Smoc^*, Cat#: NM-CKO-2112218) were genotyped by PCR using the following primers:

Forward: 5’-TGTCAGTGATTTCTGAGCTAAAGGG-3’,

Reverse: 5’- CACACAGGAATACGACCTCTCC-3’.

*Gpr75 flox/flox* mice with comparable body weights were randomly allocated into two groups. Each group received a tail vein injection of either pAAV8-TBG-NLS (control) or pAAV8-TBG-NLS-Cre-3×Flag-WPRE (OBio Technology, China) at a dose of 2×10¹¹ GC per mouse to generate hepatocyte-specific *Gpr75* knockout mice*.* After injection, mice were fed either a high-fat diet (HFD; 60% kcal from fat, 20% kcal from carbohydrates, 20% kcal from protein) for 12 weeks to induce hepatic steatosis, or a choline-deficient, L-amino acid-defined high-fat diet (CDAHFD; 60 kcal% fat, 0.1% methionine, no choline supplementation) for 8 weeks to induce MASH.

For *Gpr75* overexpression or knockdown studies, 7-week-old mice received a tail vein injection of 4x10^11^ GC of either pAAV-TBG-Gpr75-3xFlag (overexpression) or pAAV-U6-shRNA (*Gpr75*)-CBh-EGFP-WPRE (OBio Technology, China) diluted in PBS. These mice were subsequently fed a CDAHFD or HFD to investigate the role of hepatic GPR75 in MAFLD and MASH development.

To evaluate the efficacy of X201415, 8-week-old mice were randomly divided into two groups and fed a CDAHFD for 4 weeks. During the final 2 weeks, one group received daily intragastric administration of vehicle (0.5% sodium carboxymethyl cellulose, CMC-Na), while the other was treated with X201415 at 20 mg/kg per day.

**Cell culture and treatment**

HepG2 cells (Procell, China, Cat#: CL-0103) were cultured in DMEM medium with 10% FBS, and 1% penicillin-streptomycin, at 37°C with 5% CO2 in a water-saturated atmosphere. All reagent were obtained from GIBCO.

HepG2 cells were infected with rLV-CMV-GPR75-3 x flag or rLV-U6-shRNA (GPR75)-CMV-EGFP lentiviral virus (BrainVTA, China) to generate stable cell lines to overexpress or knockdown GPR75 through 0.5-2 μg/ml puromycin screening. For siRNA transfection, HepG2 cells were seeded in 12-well plates and transfected with 25 nM negative control or siRNA using Lipofectamine™ RNAiMAX (Thermo fisher, Cat#: 13778150) for 48 h according to the manufacturer's instructions. The sequences of the genes used for overexpression or knockdown are shown in Table S1.

Mouse primary hepatocyte was isolated from 8 -10 weeks old C57BL/6JNifdc mice. Briefly, liver was perfused with HBSS (no Ca^2+^, no Mg^2+^, no phenol red) for 5min and digested by collagenase IV (Sigma, Cat#: C5138) perfusion for 10 min. Subsequently, cells were dissociated and filtered through a 100-µm cell strainer. Primary hepatocyte was collected by centrifugating at 50 g x 4 ℃ for 5 mins and washed twice with DMEM and resuspended in Complete medium. 5 x 10^5^ cells per well were seeded in 12-well plates in DMEM medium containing 10% FBS, 1% penicillin-streptomycin, at 37 °C with 5% CO2. After 2 h, cells were washed with 1 x PBS. All cells were treated with palmitic acid at a dose of 200 µM for 24 h to mimic MASH model.

To explain the changes protein level of GPR75 in MASH, cells were treated with cycloheximide (100ug/mL) (MCE, USA, Cat#: HY-12320) and recombinant human CCL5 (50ug/mL) (Peprotech, USA, Cat#: 300-06-20ug) for 0 h, 2 h and 6 h after palmitic acid (200 μM) (Sigma, Cat#: P0500) stimulation. Harvested cells used to be western blot analysis.

**Immunoprecipitation-mass spectrometry (IP-MS)**

About 20 mg liver tissue obtained from mice induced with CDAHFD for 8 weeks was lysed by 1 x lysis buffer and disrupted with a glass homogenizer. The tissue extracts were centrifuged at 14,000 rpm, 4 ℃ for 10 minutes, and the supernatant was carefully aspirated 1mg protein per sample used to be incubated with rabbit IgG antibody or Gpr75 antibody at 4°C overnight. The protein A/G agarose was then added into the lysates and incubated at 4 °C for 3 h at room temperature. Collected protein A/G agarose was washed with 1x lysis buffer three times and protein was eluted with an acidic cleansing solution, followed by proteome analysis.

**Proteome analysis**

Approximately 20 mg of mouse liver was lysed with RIPA buffer to extract the protein and denatured with 8 M urea buffer. The sample was then resuspended with DTT (4 mmol/L) and alkylated with iodoacetamide solution (20 mmol/L). Trypsin was added to digest the protein (protein/enzyme, 50:1) in an incubator at 37 °C for 16 h. The eluent from the desalting cartridges was evaporated with a SpeedVac and then resuspended with water/acetonitrile (1:1, v/v). Finally, MicroLC QTOF MS and LC-MS/MS were used for proteomic analysis of mouse liver.

SCIEX M5 Microflow LC was used for on-line separation of peptides in the digestion solution of mouse liver tissue. The SCIEX Triple TOF 5600+ mass spectrometer was used in an information-dependent mode (IDA) to automatically switch between MS and MS/MS. ProteinPilot™ software (SCIEX, Concord, ON, CA, USA) and UniProtKB were used for data analysis. The specific experimental methods should be referred to the literature ^50^.

**Histological and immunohistochemistry analyses**

The liver was fixed with 4% paraformaldehyde for over 24 h and embedded in paraffin using to be analyzed for H&E, Masson, Sirus Red staining, immunocytochemistry. For Oil Red O staining, the fresh liver samples were embedded in optimal cutting temperature compound (OCT) directly and flash-frozen in liquid nitrogen, then stored at -80 ℃. All procedures were performed according to standard protocols from Wuhan Servicebio Technology or China Pharmaceutical University Pathology Platform.

**Immunofluorescence**

OCT-embedded liver samples were sectioned into 12 µm pieces after equilibration at -20 ℃, washed off the OCT with 1 x PBS, and tissue autofluorescence quenched by Wuhan Servicebio Technology. Then, the samples were blocked with 5% BSA-PBS solution at room temperature for 2 hours, followed by primary antibody incubation at 4 ℃ overnight. The corresponding secondary antibody coupled with fluorescence was reacted with the slide for 2 h at room temperature, and the nucleus was stained with DAPI dye (Beyotime, China, Cat#: C1005). Then, immunofluorescence imaging was performed using Olympus FV3000.

**Biochemical analyses of serum and livers from mice**

For lipid detection in the liver, 20 mg per sample was weighed and homogenized in anhydrous ethanol for triglycerides and total cholesterol or in saline for free fatty acids. Blood was collected from the orbital vein of mice after sacrifice and allowed to stand at room temperature for 4 hours. Serum was further collected after centrifugation at 8,000 rpm for 10 min at 4 °C and stored at – 80 °C or used immediately for AST, ALT, TG, TC and NEFA (J Njjcbio, China). All experimental procedures were performed according to the instructions provided with the kit.

**Bodipy or Nile Red staining in cell**

HepG2 cells with GPR75 overexpression or GPR75 knockdown were cultured in a confocal dish and stimulated with or without PA (200 µM) for 24 h. Harvested cells were fixed with 4% paraformaldehyde for 10 minutes at room temperature and then washed three times with 1 x PSB.

The working solution of BODIPY™ 493/503 (Invitrogen, Cat#: D3922) or Nile Red (MCE, USA, Cat#: HY-D0718) diluted to 1 µM was added to the dish and incubated for 30 mins at room temperature without light. Hoechst 33342 dye (Beyotime, China, Cat#: Cat#: C1028) was used to stain the nucleus. Imaging was performed with an Olympus FV3000.

**Co-immunoprecipitation (Co-IP)**

HepG2 cells were incubated with or without PA (200 μM) for 24 hours and lysed with IP lysis buffer containing inhibitor cocktail. Protein-containing supernatants were collected to react with primary antibodies on a tumbler at 4 ℃ overnight. The collected samples were subjected to IP with protein A/G magnetic beads (Beyotime, China, Cat#: P2179S) at room temperature for 3 hours. The beads were washed with lysis buffer and boiled with 1x SDS loading buffer before analysis by Western blotting.

**Protein isolation and Western blot analyses**

Cell or liver tissue homogenates (20 mg) were lysed in RIPA buffer (Beyotime, China) containing cocktail and protease and phosphatase inhibitors (Beyotime, China). The supernatant proteins were obtained after centrifugation at 14,000 rpm x 4℃ for 10 minutes. Nuclear, membrane or cytoplasmic proteins were separated using a protein extraction kit according to the manufacturer's instructions. All protein concentrations were measured using BCA reagent and diluted with 1 x SDS loading buffer, boiled at 100 ℃ for 5 minutes and then stored at -80 ℃. Equal amounts of protein were electrophoresed on an SDS-PAGE gel and reacted with the appropriate primary antibody after transfer to PVDF membranes. The membranes were incubated with secondary antibodies for 2 hours at room temperature. Immunoblots were detected using enhanced chemiluminescence (ECL) (Beyotime, Cat#: P0018M) followed by visualization on the ChemiDoc XRS + (Bio-Rad). Densitometric quantification of Western blots was performed using Image Lab (version 6.1). The detail information of primary antibody is shown in Supplemental information Table S3.

**Isolation and Quantitative Real-Time Polymerase Chain Reaction**

Total RNA was isolated from cells and liver tissues (10 mg) using TRIzol reagent (Vazyme, Cat# E312-01). Subsequently, 1 μg of total RNA was reverse-transcribed into cDNA using the HiScript III RT SuperMix Kit (Vazyme, Cat# R323-01). Quantitative PCR (qPCR) was performed on a CFX96 Real-Time PCR System (Bio-Rad) using 10 ng of cDNA per reaction with the ChamQ Universal SYBR qPCR Master Mix (Vazyme, Cat# Q311-02) or Custom gene qRT-PCR Quantitation Kit. Relative mRNA levels were quantified by the comparative 2^-∆∆Ct^ method. The sequences of the gene-specific primers used for PCR are shown in Supplemental information Table S4.

**DESI-MS imaging analysis**

Liver tissue samples stored at -80 °C were fixed with 10% gelatin (G108395, Aladdin, China), sectioned at 16 μm using a cryomicrotome (Leica CM1950, Leica Microsystem, Germany) at -19 ± 2 °C and mounted on positively charged slides (Thermo Fisher Scientific, Waltham, MA, USA) for storage at -80 °C. DESI MSI analysis was performed on a DESI-XEVO-G2-XS-QTOF platform (Waters, Waltham, MA, USA). Slides were dried at room temperature for 1 hour prior to analysis. Instrument calibration was performed using 0.5 mM sodium formate in 90% 2-propanol. A Harvard Apparatus Pump 11 Elite (Harvard Apparatus, Holliston, MA, USA) was used to deliver the spray solvent during data acquisition. The analysis was performed in negative mode (m/z 50-1000) with a capillary voltage of 3.5 kV, an ion source temperature of 130 °C and a nitrogen pressure of 0.45 MPa. The solvent (ACN: water, 80:20 v/v) contained 200 pg/μl leucine enkephalin, delivered at 2 μl/min. Mass spectra were processed using Mass Lynx 4.1 and HDI Imaging software, and Progenesis QI was used for isotopic interference elimination and identification.

**S Table 1.** The sequences of the genes used for knockdown. Related to methods.

| GENES | SEQUENCES | COMPANY |
| --- | --- | --- |
| mouse shRNA (Gpr75) | 5’-TGTATAGGAACCAGAATTATTC  AAGAGATAATTCTGGTTCCTATACA -3’ | OBio Technology, China |
| mouse shRNA (Vps35) | 5’- CTTAGTAATGTTCTGGATTAT-3’ | OBio Technology, China |
| mouse shRNA (Gnai2) | 5’- GCATTGCACAGAGTGACTA -3’ | OBio Technology, China |
| human shRNA (Gpr75) | 5’-GAACCTGTCCTTCTGTGACCTTTCA  AGAGAAGGTCACAGAAGGACAGGTTC-3’ | BrainVTA, China |
| human siRNA (Vps35) | 5’-CCTCCAGACTTTGAATCCTTT-3’ | Genepharma, China |
| mouse siRNA (Gnai2) | 5’- GACCATCTGCTTCCCTGAG-3’ | Genepharma, China |

**S Table 2.** Liver biopsy patient information.

| DIAGNOSIS | SEX | AGE |
| --- | --- | --- |
| Normal | Female | 42 |
| Normal | Male | 30 |
| Normal | Male | 23 |
| Normal | Male | 40 |
| Normal | Male | 34 |
| Normal | Female | 40 |
| Normal | Male | 23 |
| Steatohepatitis | Male | 68 |
| Mild steatosis | Female | 64 |
| Moderate steatosis | Male | 62 |
| Severe steatosis | Male | 57 |
| Steatohepatitis | Female | 57 |
| Steatohepatitis | Male | 70 |
| Stage F2 fibrosis | Female | 63 |
| Stage F2 fibrosis | Female | 57 |
| Stage F2 fibrosis | Male | 68 |
| Stage F3 fibrosis | Male | 53 |
| Stage F3 fibrosis | Male | 75 |
| Stage F4 fibrosis | Male | 64 |

Fibrosis was assessed using the Ishak staging criteria.

**S Table 3.** The information about primary antibody. Related to methods.

| Antibodies | Company | Identifier |
| --- | --- | --- |
| Anti-GPR75 for WB | Bioworld, China | Cat#: BS5752 |
| Anti-GPR75 for IF | Proteintech, USA | Cat#: 17972-1-AP |
| Anti-GAPDH for WB | Abcam, UK | Cat#: ab181602 |
| Anti-α-SMA for WB/IF | Abcam, UK | Cat#: ab124964 |
| Anti-Albumin for IF | Proteintech, USA | Cat#: 66051-1-Ig |
| Anti-CD31 for IF | Abcam, UK | Cat#: ab7388 |
| Anti-F4/80 for IF | Abcam, UK | Cat#: ab6640 |
| Anti-COl1A for WB | Abcam, UK | Cat#: ab21286 |
| Anti-GNAI2 for WB/IP | Proteintech, USA | Cat#: 11136-1-AP |
| Anti-GNAI2 for WB/IP/IF | Proteintech, USA | Cat#: 67007-1-Ig |
| Anti- PKAc for WB | Proteintech, USA | Cat#: 27398-1-AP |
| Anti-phospho-PKAc (Thr197) for WB | Cell Signaling, USA | Cat#: 4781 |
| Anti-phospho-PKAc Substrate for WB | Cell Signaling, USA | Cat#: 9624 |
| Anti-SREBP-1c for WB | Invitrogen, USA | Cat#: MA5-16124 |
| Anti-VPS35 for WB/IP/IF | Proteintech, USA | Cat#: 10236-1-AP |
| Anti-VPS35 for WB/IP/IF | SantaCruz, USA | Cat#: sc-374372 |
| Anti-LAMP1 for IF | Abcam, UK | Cat#: ab25630 |
| Anti-LAMIN B1 for WB | Abcam, UK | Cat#: ab25630 |
| Anti-Na, K-ATPase for WB | Cell Signaling, USA | Cat#: 3010 |
| Anti-UBN2E(UBC13) for WB/IP | Proteintech, USA | Cat#: 10243-1-AP |
| Anti-Flag for WB/IP | Cell Signaling, USA | Cat#: 14793 |
| Anti-Flag for WB/IP | Sigma, USA | Cat#: F1804 |
| Anti-HA for WB/IP | Cell Signaling, USA | Cat#: 3724 |
| Goat anti-Mouse IgG HRP | Bioworld, China | Cat#: BS12478 |
| Goat anti-Rabbit IgG HRP | Bioworld, China | Cat#: BS13278 |
| Goat anti-Rat IgG, Alexa Fluor™ 647 | Thermofisher, USA | Cat#: A-21247 |
| Donkey anti-Mouse IgG, Alexa Fluor™ 488 | Thermofisher, USA | Cat#: A-21202 |
| Goat Anti-Rabbit IgG, YS Fluor™ 594 | Yeasen, China | Cat#: 33112ES60 |
| Donkey anti-Rabbit IgG, Alexa Fluor™ 555 | Thermofisher, USA | Cat#: A-31572 |
| Donkey Anti-Mouse IgG, Cy™5 | Laboratories, USA | Cat#: 715-175-150 |

**S Table 4.** Primers (mice) for qPCR. Related to methods.

| **Genes and Sequences** | **Company** | **Identifier** |
| --- | --- | --- |
| *Gapdh* forward: 5’-CACTGAGCATCTCCCTCACA -3’ | Sangon Biotech, China | NA |
| *Gapdh* reverse: 5’-GTGGGTGCAGCGAACTTTAT -3’ | Sangon Biotech, China | NA |
| *Gpr75* forward: 5’-CTCAGGCTTCGTCATCATGTC -3’ | Sangon Biotech, China | NA |
| *Gpr75* reverse: 5’-AGGGTAAGGAGCAAGATGCAG -3’ | Sangon Biotech, China | NA |
| *Vps35* forward: 5’-TTGGTAGAAATGTGCCGTGGTGT-3’ | Sangon Biotech, China | NA |
| *Vps35* reverse: 5’-CATCCGCACCCAGAGCTTATTCA-3’ | Sangon Biotech, China | NA |
| *Gnai2* forward: 5’- CAGATCGACTTTGCTGATCCC-3’ | Sangon Biotech, China | NA |
| *Gnai2* reverse: 5’-TAAGCGGCTGAGTCATTGAGC-3’ | Sangon Biotech, China | NA |
| *Srebp-1c* forward: 5’-CATGGACGAGCTGGCCTTC-3’ | Sangon Biotech, China | NA |
| *Srebp-1c* reverse: 5’-ACTGTCTTGGTTGTTGATGAGC-3’ | Sangon Biotech, China | NA |
| *Fasn* forward: 5’-TAGAACCTCCAGTCGTGAAACCA-3’ | Sangon Biotech, China | NA |
| *Fasn* reverse: 5’-ATCTCATCTATCTTGCCCTCCTTG -3’ | Sangon Biotech, China | NA |
| *Acc1* forward: 5’-GCTAACCCAACTCAGCAAGACC -3’ | Sangon Biotech, China | NA |
| *Acc1* reverse: 5’- CACCTGGTTGCTGTGGTAAAAA -3’ | Sangon Biotech, China | NA |
| *Scd1* forward: 5’-TTCCCTCCTGCAAGCTCTAC-3’ | Sangon Biotech, China | NA |
| *Scd1* reverse: 5’-CAGAGCGCTGGTCATGTAGT-3’ | Sangon Biotech, China | NA |
| *Gpat1* forward: 5’-CCATTGTGGAGGATGAAGTG-3’ | Sangon Biotech, China | NA |
| *Gpat1* reverse: 5’-TGGATCGTGCCAGATAGGGA-3’ | Sangon Biotech, China | NA |
| *Dgat1* forward: 5’-GCCCCTGGACAACACAGACT-3’ | Sangon Biotech, China | NA |
| *Dgat1* reverse: 5’-CCATTGTGGAGGATGAAGTG-3’ | Sangon Biotech, China | NA |
| *Tnfα* forward: 5’-AGCCGATGGGTTGTACCTTG-3’ | Sangon Biotech, China | NA |
| *Tnfα* reverse: 5’-GTGGGTGAGGAGCACGTAGTC-3’ | Sangon Biotech, China | NA |
| *Il-1β* forward: 5’-GCAACTGTTCCTGAACTCAACT-3’ | Sangon Biotech, China | NA |
| *Il-1β* reverse: 5’-ATCTTTTGGGGTCCGTCAACT-3’ | Sangon Biotech, China | NA |
| *Il-6* forward: 5’-GATGCTACCAAACTGGATATAATC-3’ | Sangon Biotech, China | NA |
| *Il-6* reverse: 5’-GGTCCTTAGCCACTCCTTCTGTG-3’ | Sangon Biotech, China | NA |
| *Mcp1* forward: 5’- GGGCCTGCTGTTCACAGTT -3’ | Sangon Biotech, China | NA |
| *Mcp1* reverse: 5’- CCAGCCTACTCATTGGGAT -3’ | Sangon Biotech, China | NA |
| *Col1a1* forward: 5’-GAACTGGACTGTCCCAACCC-3’ | Sangon Biotech, China | NA |
| *Col1a1* reverse: 5’-TTGGGTCCCTCGACTCCTAC-3’ | Sangon Biotech, China | NA |
| *Acta2* forward: 5’- CACCCAGCATGAAGATCAAG -3’ | Sangon Biotech, China | NA |
| *Acta2* reverse: 5’-TCTGCTGGAAGGTAGACAGCGAAG-3’ | Sangon Biotech, China | NA |
| *Tgfβ* forward: 5’-AGGAGACGGAATACAGGGCT -3’ | Sangon Biotech, China | NA |
| *Tgfβ* reverse: 5’-CCACGTAGTAGACGATGGGC -3’ | Sangon Biotech, China | NA |
| *Pdgfβ* forward: 5’-TGCACAGAGACTCCGTAGATGA -3’ | Sangon Biotech, China | NA |
| *Pdgfβ* reverse: 5’-CACTCGGCGATTACAGCAGG -3’ | Sangon Biotech, China | NA |
| *Ctgf* forward: 5’-AGAACTGTGTACGGAGCGTG -3’ | Sangon Biotech, China | NA |
| *Ctgf* reverse: 5’-GGTGCACCATCTTTGGCAGT -3’ | Sangon Biotech, China | NA |
| *Timp1* forward: 5’-ACTCGGACCTGGTCATAAGGG -3’ | Sangon Biotech, China | NA |
| *Timp1* reverse: 5’-CGCTGGTATAAGGTGGTCTCGT -3’ | Sangon Biotech, China | NA |
| *Cd36* forward: 5’-GACTGGGACCATTGGTGATGA -3’ | Sangon Biotech, China | NA |
| *Cd36* reverse: 5’-AAGGCCATCTCTACCATGCC -3’ | Sangon Biotech, China | NA |
| *Fabp1* forward: 5’-TGGTCCGCAATGAGTTCACCCT -3’ | Sangon Biotech, China | NA |
| *Fabp1* reverse: 5’-CCAGCTTGACGACTGCCTTGACTT -3’ | Sangon Biotech, China | NA |
| *Pparα* forward: 5’-AGAGCCCCATCTGTCCTCTC -3’ | Sangon Biotech, China | NA |
| *Pparα* reverse: 5’-ACTGGTAGTCTGCAAAACCAAA -3’ | Sangon Biotech, China | NA |
| *Cpt-1α* forward: 5’-CTCCGCCTGAGCCATGAAG -3’ | Sangon Biotech, China | NA |
| *Cpt-1α* reverse: 5’-CACCAGTGATGATGCCATTCT-3’ | Sangon Biotech, China | NA |
| *Cpt-2* forward: 5’-CCTGCTCGCTCAGGATAAACA -3’ | Sangon Biotech, China | NA |
| *Cpt-2* reverse: 5’- GTGTCTTCAGAAACCGCACTG-3’ | Sangon Biotech, China | NA |
| *Pgc-1α* forward: 5’-TATGGAGTGACATAGAGTGTGCT -3’ | Sangon Biotech, China | NA |
| *Pgc-1α* reverse: 5’-CCACTTCAATCCACCCAGAAAG-3’ | Sangon Biotech, China | NA |
